# PET/CT imaging of tuberculosis lung lesions in marmosets treated with different drug regimens aligns with human clinical outcomes

**DOI:** 10.1126/scitranslmed.ado9383

**Published:** 2026-01-07

**Authors:** Talia Greenstein, Laura E. Via, Mariana Pereira Moraes, Danielle M. Weiner, Emmanuel K. Dayao, April M. Walker, Ayan Abdi, Joel D. Fleegle, Felipe Gomez, Katelyn M. Repoli, Michael J. Woodcock, Helena I.M. Boshoff, Oluwaseun Egbelowo, Kamunkhwala Gausi, Paolo Denti, Firat Kaya, Matthew Zimmerman, Eric L. Miller, Véronique A. Dartois, Clifton E. Barry, Bree B. Aldridge

**Affiliations:** 1 Department of Molecular Biology and Microbiology, Tufts University School of Medicine; Boston, MA, United States.; 2 Graduate School of Biomedical Sciences, Tufts University School of Medicine; Boston, MA, United States.; 3 Tuberculosis Research Section, Laboratory of Clinical Immunology and Microbiology, NIAID, NIH; Bethesda, MD, United States.; 4 Tuberculosis Imaging Program, Division of Intramural Research, NIAID, NIH, Bethesda, MD, United States.; 5 Division of Clinical Pharmacology, Department of Medicine, University of Cape Town; Cape Town, South Africa.; 6 Center for Discovery and Innovation, Hackensack Meridian Health; Nutley, NJ, United States.; 7 Department of Electrical and Computer Engineering, Tufts University School of Engineering; Medford, MA, United States.; 8 Hackensack Meridian School of Medicine, Department of Medical Sciences; Nutley, NJ, United States.; 9 Institute of Infectious Disease and Molecular Medicine, Faculty of Health Sciences, University of Cape Town; Cape Town, South Africa.; 10 Department of Biomedical Engineering, Tufts University School of Engineering; Medford, MA, United States.; 11 Stuart B. Levy Center for Integrated Management of Antimicrobial Resistance; Boston, MA, United States.

## Abstract

Early bactericidal activity and time to sputum conversion are well-established study endpoints in both preclinical models and clinical trials for testing drug regimens for pulmonary tuberculosis (TB). The development and optimization of new, treatment-shortening drug regimens for TB has been challenged by disparities between these study endpoints and non-relapsing cure. We hypothesized that using lung lesions revealed by 2-deoxy-2-[^18^F]fluoro-D-glucose (FDG)-positron emission tomography/computed tomography (PET/CT) imaging could help to interpret treatment efficacies and better understand clinical treatment outcomes. Radiographic changes in lung lesions were measured using PET/CT imaging in a cohort of infected marmosets, which were divided into 22 treatment arms (including monotherapies and combination drug treatments) for two months. We used unsupervised clustering to define multivariate treatment response profiles that combined quantitative changes in radiographic pathology and terminal bacterial burden per lung lesion to inform lesion-level responses to drug treatments. These drug response profiles not only aligned with known clinical outcomes but also provided lesion-level insights into clinical successes and failures. We found that the inferiority of the four-month moxifloxacin-rifampicin-pyrazinamide-ethambutol regimen compared to the six-month standard of care for individuals with lung cavitary TB could be predicted. The marmoset response profiles were matched to their respective histopathological classifications at necropsy and successfully distinguished cavitary granulomas that responded to treatment from cavitary granulomas that failed to improve or worsened after the first month of treatment. Our findings indicate that a combination of quantitative PET/CT measures is more informative of TB treatment outcomes than bacterial burden.

## One Sentence Summary

Longitudinal PET/CT imaging results in marmosets align with clinical tuberculosis treatment outcomes.

## Accessible Summary

Tuberculosis treatment outcomes are influenced by variations in lesion types and severity. Development of more effective treatment regimens requires tools that interrogate lesion-level differences in drug treatment response to bridge the gap between preclinical models and clinical outcomes. In this study, we analyzed PET/CT changes in response to different drug regimens in tuberculosis lesions in infected marmosets. We found that multivariate profiles of marmoset lesion characteristics aligned with clinical outcomes and were more informative of responses to treatment than any single, measurable outcome alone.

## INTRODUCTION

As we enter a new era of drug candidates and combinations for tuberculosis (TB) treatment, there is an urgent need for meaningful clinical biomarkers of treatment success. Human TB is an extremely heterogeneous disease with multiple types of lung lesions that exist simultaneously within a single patient with active disease (1, 2). These lesion types present distinct biological environments for the bacteria and respond differentially to drug treatment because of diverse states of bacterial physiology and differential drug penetration (3–5). The standard of care for drug-sensitive TB is two months (“intensive phase”) of isoniazid (H), rifampin (R), pyrazinamide (Z), and ethambutol (E), followed by four to seven months of isoniazid and rifampin (“continuation phase”). The current “one-size-fits-all” approach is implemented with the knowledge that we overtreat the majority of patients to cure the small percentage of patients with disease that would otherwise relapse after treatment is discontinued (6, 7). The primary endpoints from clinical trials rely on the enumeration of live bacilli within expectorated sputum samples from patients with TB, as the most common biomarker, because of ease of sampling. However, 70–90% of patients have no bacteria left in their sputum after the first two months of chemotherapy, despite not having achieved a relapse-free status. Analysis of sputum culture conversion as a surrogate endpoint for relapse has revealed that it is not reliable across different populations and treatment regimens (8). For example, the REMox TB trial tested the treatment-shortening potential of four months of moxifloxacin-rifampicin-pyrazinamide-ethambutol (MRZE) against the six-month standard of care. The trial resulted in more unfavorable outcomes (treatment failure or relapse by 18 months) in the experimental arm than in the control arm despite earlier sputum conversion in the experimental arm (9). Disparities between treatment outcomes create confusion regarding the predictive power of preclinical models.

We previously reported that changes in 2-deoxy-2-[^18^F]fluoro-D-glucose (FDG)-positron emission tomography (PET)/computerized tomography (CT) in patients with multidrug-resistant (MDR) TB disease and drug-susceptible (DS) TB disease correlated with non-relapsing cure (10, 11). The radiologic features and sputum conversion were evaluated as separate entities in these studies. We sought to quantitatively link these measures to identify relationships between PET/CT pathology, bacterial burden, and non-relapsing cure.

Simple murine models of TB do not replicate this heterogeneous pathology and although more advanced murine models do, they only replicate a subset of the pathology seen in a typical TB patient about to initiate chemotherapy (12). Non-human primates do develop advanced pathology, including cavitary lesions, and are a good predictor of clinical outcomes for both single agents and combination therapies (13). Though per-lesion bacterial burden is a powerful and quantitative endpoint, it can only be measured at necropsy. Longitudinal radiological studies allow for real-time assessment of changes in TB-associated pathology. Although non-human primates recapitulate human disease quite well, each animal often presents with large numbers of individual lesions of different types, which presents an analytical challenge given the complexity of the multidimensional data acquired.

We hypothesized that a multivariate description of lesion outcomes based on PET/CT data and per-lesion bacterial burden would be a more informative measure of treatment outcome than any one metric. Here, we conducted a lesion-specific drug response study in marmosets (*Callithrix jacchus*) and quantified FDG-PET/CT pathology over time and terminal bacterial burden as the treatment outcomes across 22 drug treatment arms. We used unsupervised clustering to define categories of lesion-specific responses to TB treatment based on PET/CT pathology and bacterial burden. We found that this combination of metrics aligned better with clinical expectations than any individual metric and thus could be used to understand treatment success or failure at the lung lesion level.

## RESULTS

### Description of marmoset experiments and data readouts

Marmosets were infected with *Mycobacterium tuberculosis* (Mtb) H37Rv via aerosol as previously described (13, 14). Between six and seven weeks after infection, treatment was started (Tables 1, table S1). We observed improvement in signs of disease (e.g., weight loss, inappetence, activity level) within two to four weeks of treatment for most treatment groups. PET/CT scans were taken at the start of treatment and every two weeks thereafter until eight weeks of treatment, after which the animals were humanely sacrificed. A PET/CT image-guided necropsy was performed for each animal, with organ weights and samples of each lung lesion collected for bacteria enumeration and histopathological classification.

Marmoset responses to treatment were monitored by PET/CT as previously described (13). In brief, structures in the lung were demarcated based on radiodensity (measured in Hounsfield Units, HU), where lesions were defined as structures with radiodensity greater than −500 HU. PET/CT scans were co-registered to allow for tracking of each lesion across all scans. Relevant PET and CT features were quantified within each specified region of interest (ROI, i.e., lesion). The mean and standard deviation of density across all voxels within an ROI were extracted for each lesion over time. Areas with radiodensity between −100 and +200 HU were designated as “hard density,” and areas between −500 and −100 HU were designated as “soft density.” These areas were converted to volumes within each lesion. Hard and soft density volumes inform the quantity (i.e., size) and severity of the lesions. The mean standardized uptake value (SUV) of FDG within an ROI was extracted from the co-registered PET scan. Total lesion glycolysis (TLG) was quantified by identifying ROI voxels in the CT and computing total SUV activity in these voxels in subsequent co-registered PET scans.

### Unsupervised clustering defines a multivariate description of complete treatment outcomes

We previously reported that PET/CT changes correlated with non-relapsing cure in TB patients with higher sensitivity than sputum conversion (10, 11). To understand the relationship between PET/CT measures and bacterial burden in marmosets, we explored correlations between the PET/CT features and terminal bacterial burden after two months of drug treatment. We found that total lung bacterial burden was not correlated with any individual PET/CT biomarker or change in individual PET/CT biomarkers at two months, suggesting that these biomarkers contain non-redundant information (Fig. S1). We observed that treatment outcomes by PET, CT, and bacterial burden measures were disparate, with no correlation from metric to metric. For example, the ranking of treatment arms by total lung bacterial burden differed from the ranking by change (i.e., improvement) in total lung hard density volume. The lack of correlation between PET/CT features and bacterial burden and disparity in the rank-ordering of treatment arms was also observed at the lesion level (Fig. S2).

Because single PET, CT, and bacterial burden measures did not correlate, we hypothesized that they could be combined in a multivariate signature that would serve as a single, interpretable measure of treatment outcome that would inform clinical outcomes. We focused on lesion-level treatment outcomes because independent lesion evolution and resolution have been reported in PET/CT studies (10, 15, 16). We compiled a multivariate profile that included features describing lesion characteristics from PET/CT after end of therapy (EOT), changes in PET/CT features from the beginning of treatment to EOT (eight weeks total), and per-lesion bacterial burden at EOT across all lesions from all 22 treatment arms. We sought to characterize groups of lesions with similar feature profiles to identify unique lesion evolution patterns in response to drug treatment. We used unsupervised clustering to identify nine distinct lesion populations based on similar response profiles (Fig. S3).

**Figure 1.**
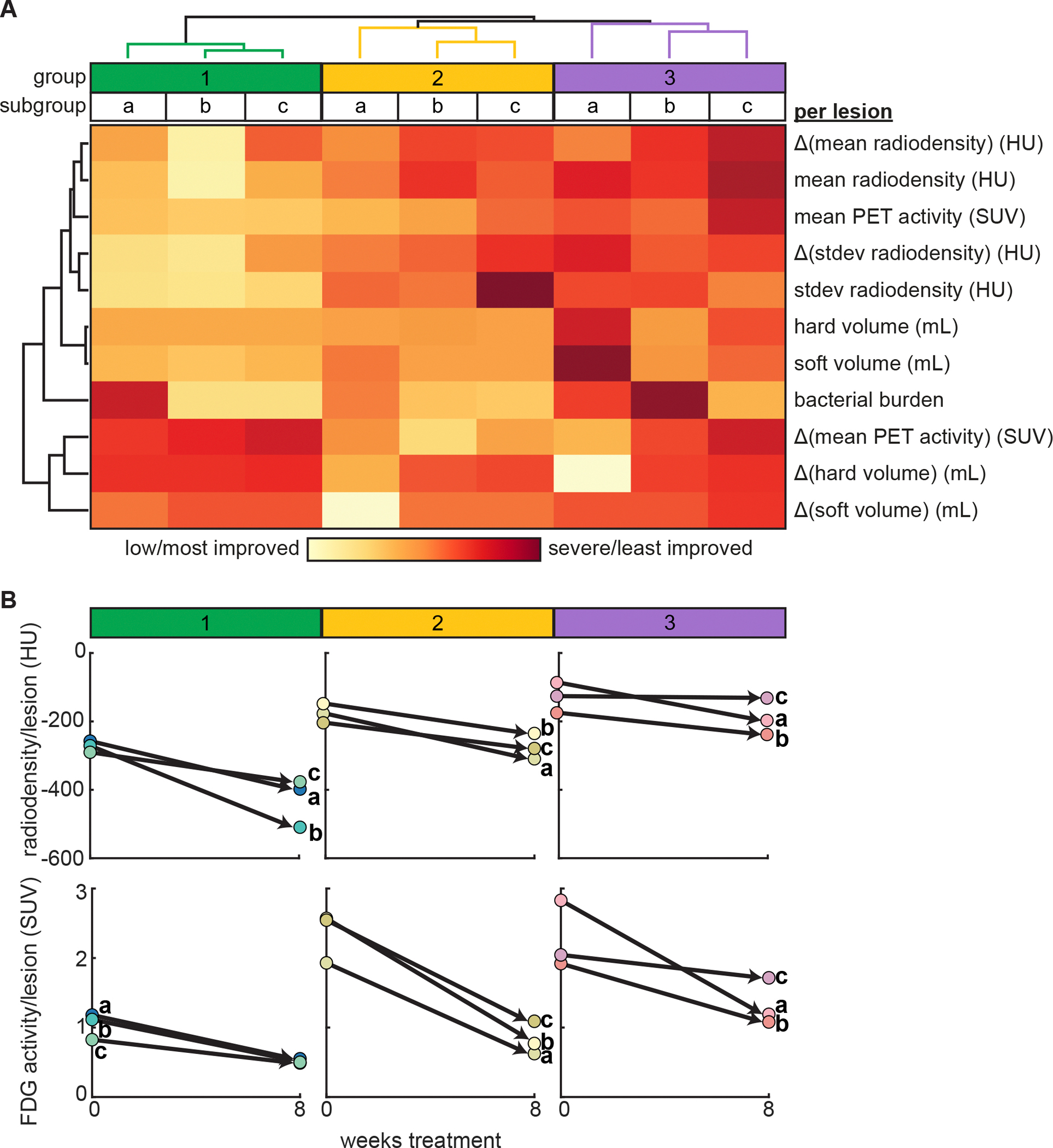
Characteristics of each lung lesion cluster and cluster distribution across all lesions. **(A)** Heatmap of hierarchical clustering of median feature values across the clusters from Leiden community detection. Three groups (1, 2, 3) of similar clusters are labeled from the hierarchical clustering, and clusters are labeled (a, b, c) within each group. Feature values were scaled for visualization. The severe/low color scale is relative to the values in this visualization (not absolute). Δ refers to changes in a feature from the start (0 weeks) to end of treatment (EOT; 8 weeks). **(B)** Median values for mean radiodensity per lesion (CT measure, top) and mean FDG activity per lesion (PET measure, bottom) at the start (0 weeks) and end (8 weeks) of treatment for each cluster (labeled a-c) within each group (labeled 1–3).

To understand the unique feature profiles that define each lesion cluster, we evaluated the median feature values within each cluster and performed hierarchical clustering on the aggregate features across the nine clusters (Fig. 1A, fig. S4, Table S2). Three groups (named 1, 2, and 3) of lesion clusters emerged based on the similarity of features across the member clusters. The group numbers were assigned to indicate order from lowest to highest severity based on the aggregate features. Each group had broad feature commonalities with specific feature differences that distinguished their member clusters. Group 1 was characterized by the lowest PET/CT feature values after EOT and relatively little improvement in PET/CT pathology during that time. Group 1 lesions generally had the lowest pathology (e.g., radiodensity and PET activity) at the start of treatment relative to the other groups (Fig. 1B, fig. S5). Within group 1, lesions in clusters 1b and 1c were characterized by low bacterial burden, and lesions in cluster 1a were characterized by relatively higher bacterial burden. The majority of lesions from cluster 1a were located on or adjacent to bronchi, which likely affected drug delivery to those lesions, making them harder to clear. Group 2 lesions were characterized by moderate or varied (i.e., not uniformly severe or low) PET/CT pathology after eight weeks of treatment and mid/low bacterial burden. Within that group, lesions in clusters 2b and 2c were characterized by improvement (decrease) in mean PET activity, and lesions in cluster 2a were characterized by the most improvement (decrease) in hard and soft volume (Fig. 1B, fig. S5). Group 3 lesions were characterized by the most severe PET/CT features after EOT. Within that group, lesions in clusters 3a and 3b were characterized by high bacterial burden, and lesions in cluster 3c were characterized by low bacterial burden. Cluster 3a was also distinguished by improvement in mean PET activity and hard volume (Fig. 1B, fig. S5). Lesions in groups 2 and 3 tended to have greater radiodensity and PET activity than lesions in group 1 at the start of treatment, indicating that lesions that ultimately clustered in groups 2 and 3 based on terminal PET/CT features and changes in those features had more severe pathology at the start of treatment (Fig. S6). Lesions in groups 2 and 3 were distinguished by detected changes in PET/CT pathology over eight weeks of treatment and differences in terminal PET/CT pathology and bacterial burden (Fig. 1B). This analysis, therefore, separated “responder” and “non-responder” lesions that started treatment with severe pathology detected by PET/CT.

**Figure 2.**
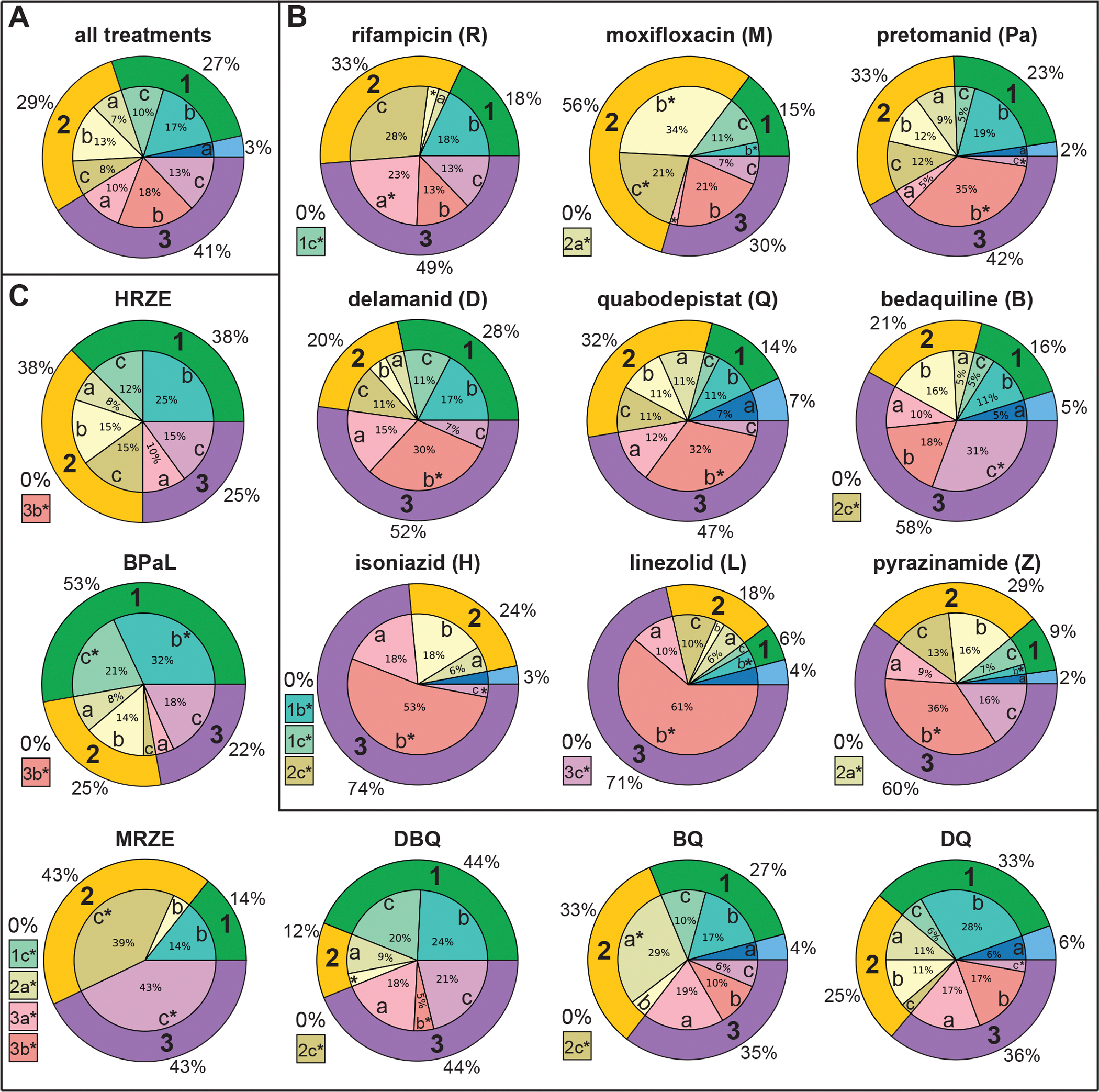
Distribution of complete treatment clusters across lung lesions. Inner pie charts represent the analysis of the distribution of clusters from the complete treatment (two months) across individual drug treatments. Cluster groups are indicated in the outer pie charts: (1) Low bacterial burden and low/improved PET/CT pathology; (2) low or varied bacterial burden and moderate/improved PET/CT pathology; (3) severe/worsened PET/CT pathology and high bacterial burden, except 3c. Outer wedge colors correspond to those in Figure 1. *Indicates significant deviation (95% confidence interval) from the expected (average) number of lesions in a specific cluster by adjusted residual analysis following the chi-squared test for homogeneity (p < 0.001). **(A)** Distribution of the clusters across all lung lesions from all drug treatments, representing the average distribution across drug treatments (n = 1193 lesions). **(B)** Distribution of the clusters across all lesions for each of the monotherapy treatment arms. Monotherapies are ordered by ranking from total lung colony-forming units (CFU) (Figure S1A). **(C)** Distribution of the clusters across all lesions for specific combination treatment arms. Boxes to the left of each pie chart in panels B and C indicate significant underrepresentation (complete absence) from specified clusters. Unlabeled inner slices are <5% of the total for that drug treatment. The other seven treatment arms not shown in this figure are in Fig. S10.

Untreated marmosets infected with the same infectious dose of Mtb would not survive beyond 8–10 weeks; therefore, this study was limited to drug-treated animals. To evaluate the robustness of the clustering results, we performed a leave-one-out analysis whereby we re-clustered the lesions using Leiden community detection 22 times, each time skipping all lesions from one treatment arm. We observed similar patterns across the clusters in all iterations, demonstrating that the clustering results were not strongly dependent on any specific treatment arm (Fig. S7). The lesion modeling was also robust to multiple clustering algorithms and levels of resolution in community detection (Fig. S8, S9). In the absence of untreated lesions, we used the distribution of lesions from all animals across the different clusters as a baseline to compare individual drug treatments to all treatments (Fig. 2A). In this analysis, 41% of the total lesions from all treatment arms were represented in the high PET/CT pathology group 3 (purple), while the others were split between the low PET/CT pathology group 1 (27% in 1b and 1c, green), moderate/varied group 2 (29%, yellow), and the low pathology, high burden cluster (3% in 1a, blue). Comparison of cluster distribution within individual treatment arms to the distribution across all treatments provided a new way to interpret lesion-specific treatment outcomes. We hypothesized that deviations from the distribution across all treatments (the “baseline”) could be used to identify drug treatments that resulted in a greater abundance of improved lesions and, conversely, fewer severe lesions. For example, we evaluated the distribution of clusters across all lesions in each of the monotherapy treatment arms (Fig. 2B). Across all lesions within each of the nine monotherapy treatment arms, group 3 lesions were significantly (adjusted residuals [adj.res] > 1.96) overrepresented in eight out of nine arms compared to all treatments, and group 1 or 2 lesions were underrepresented (adj.res < −1.96, using adjusted residual analysis after chi-squared test for homogeneity, significance thresholds ±1.96 and ±2.58 corresponding to 95% and 99% confidence intervals, respectively). The abundance of severe pathology lesions paired with the lack of low and moderate pathology lesions suggested that rather than induce resolution or improvement with drug treatment, most monotherapies failed to adequately target lesions with severe PET/CT pathology at the start of treatment, resulting in continued severe pathology and, in some cases, high bacterial burden at EOT.

### Lesion-specific treatment outcomes align with efficacy of clinical standards of care

We next evaluated the distribution of clusters across all lesions in animals treated with isoniazid-rifampicin-pyrazinamide-ethambutol (HRZE), the clinical standard of care for drug susceptible-TB (Fig. 2C). Though HRZE-treated lesions were overrepresented in the low pathology/low bacterial burden clusters 1b and 1c, the deviation from all treatments was not statistically significant. There was a significant underrepresentation (adj.res < −2.58) of HRZE-treated lesions in cluster 3b (high pathology, high bacterial burden). The absence of HRZE-treated lesions in cluster 3b (high pathology, high bacterial burden) suggested that HRZE reduced bacterial burden in lesions with severe pathology. The lack of difference between the proportion of HRZE-treated lesions in clusters 3a and 3c and the overall proportion of lesions in those clusters suggested that HRZE did not improve PET/CT pathology in lesions with severe pathology more or less than other treatment arms in this study. Taken together, these distributions indicated that though HRZE effectively reduced the bacterial burden, it was not markedly more effective at reducing severe pathology compared to other treatment arms in this study.

We next evaluated the distribution of clusters across all lesions in animals treated with bedaquiline-pretomanid-linezolid (BPaL), the approved drug regimen for multidrug-resistant TB (Fig. 2C). BPaL-treated lesions were significantly overrepresented (adj.res > 2.58) in the low pathology, low bacterial burden clusters from group 1 (1b and 1c) and underrepresented in lesions from clusters 2c and 3a (not statistically significant). As with HRZE, there was a significant underrepresentation (adj.res <−2.58) of BPaL-treated lesions in cluster 3b (high pathology, high bacterial burden). The distribution of BPaL-treated lesions across the different clusters suggested that BPaL simultaneously resolved more of the moderate and low pathology lesions than other treatments, resulting in a greater percentage of lesions in group 1 and fewer in groups 2 and 3, and effectively reduced bacterial burden in lesions with severe PET/CT pathology. These findings support the observed clinical efficacy of BPaL and suggest that it may outperform HRZE in the resolution of lesions with low-to-moderate PET/CT pathology.

Total lung bacterial burden after two months of treatment with HRZE or BPaL was low (Fig. S1A); however, after two months of treatment, 25% of HRZE-treated lesions and 22% of BPaL-treated lesions presented with severe PET/CT pathology (represented in group 3). 15% of HRZE-treated and 18% of BPaL-treated lesions were in cluster 3c, characterized by low bacterial burden and severe PET/CT pathology, with minimal improvement or worsening of pathology. In a previous clinical PET/CT study, we reported that an increase or minimal change in PET activity at two months of treatment was associated with treatment failure (10). Therefore, we hypothesized that in the absence (or reduction) of lesions in the other clusters of group 3, the lesions in cluster 3c represent a potential niche for treatment failure or relapse. We observed that not all HRZE- and BPaL-treated animals had lesions in cluster 3c; of the five HRZE-treated animals, one animal had three lesions in cluster 3c, and three animals had one lesion each in cluster 3c after two months of treatment. Of the five BPaL-treated animals, one animal had ten lesions in cluster 3c and two animals had 1–2 lesions each in cluster 3c after two months of treatment. This non-uniform resolution of severe pathology lesions in some HRZE- or BPaL-treated animals but not others may reflect inter-animal heterogeneity in responses.

### Lesion-specific treatment outcomes in marmosets predict the failure of MRZE

In the REMox TB clinical trial, unfavorable outcomes (including relapse and treatment failure) occurred earlier and more frequently in the MRZE arm compared to the control arm (HRZE), despite earlier sputum conversion with MRZE (9). Given the disparate ranking of MRZE based on single-metric treatment outcomes in the marmoset (Fig. S1A,D), we asked if the multivariate response profiles of MRZE-treated lesions could identify poor outcomes at the lesion level that would explain the clinical outcome. We evaluated the distribution of clusters across all lesions in animals treated with MRZE and observed striking differences from the other higher-order treatment arms (Fig. 2C). MRZE-treated lesions were significantly overrepresented (adj.res > 2.58) in cluster 3c from the severe pathology group (group 3), which is characterized by lesions that presented with severe PET/CT pathology at the start of treatment that did not resolve or improve within eight weeks of treatment, despite the reduction of bacterial burden. As with the other higher-order combinations, there was a significant underrepresentation (adj.res <−2.58) of MRZE-treated lesions in cluster 3b (high pathology, high bacterial burden). Unlike the other higher-order treatment groups, however, there was significant underrepresentation (adj.res <−1.96) of MRZE-treated lesions from low and moderate PET/CT pathology clusters in groups 1 and 2. These data suggested that although treatment with MRZE reduced bacterial burden, such that MRZE-treated lesions were not represented in the high bacterial burden clusters, the reduction in bacterial burden did not coincide with improvement in PET/CT pathology. We hypothesized that lesions with severe pathology (as detected by PET/CT) were more effectively targeted by BPaL and HRZE than by MRZE, and this improvement was reflected in the underrepresentation in group 3 lesions and overrepresentation in group 1 lesions observed in BPaL and HRZE-treated animals (which did not occur in MRZE-treated animals). The failure of MRZE to effectively target and reduce the severe pathology lesions culminated in a different treatment outcome for MRZE-treated individuals compared to HRZE- or BPaL-treated individuals, that is, a higher incidence of treatment failure and relapse. We observed that the response profiles of MR-treated lesions indicated greater efficacy in the reduction of pathology than in MRZE-treated lesions, suggesting potential antagonism in the higher-order combination (Fig. S10). MRZE and MR treatments resulted in similarly low per-lesion bacterial burden (Fig. S1); the antagonism was specifically in the reduction of pathology, suggesting that this antagonism may be against immune activity rather than drug action.

### Marmoset model predicts potential reduction of PET/CT pathology by quabodepistat combined with bedaquiline

Quabodepistat (OPC-167832) is an investigational agent for tuberculosis treatment that inhibits the DprE1, an essential enzyme for cell wall biosynthesis in mycobacteria. Quabodepistat (Q) demonstrates potent antimycobacterial activity *in vitro* and *in vivo* and is currently in clinical trials in combination with bedaquiline (B) and delamanid (D) (17–19) (NCT05221502). We evaluated the distribution of clusters across all lesions in animals treated with quabodepistat in pairwise combinations with bedaquiline or delamanid and the three-way combination DBQ. BQ-treated lesions were significantly overrepresented (adj.res > 2.58) in cluster 2a, which represented lesions that started treatment with severe PET/CT pathology that demonstrated marked improvement at EOT, and were underrepresented in group 3 (35% total compared to 41% average, results not statistically significant), which represented lesions with the most severe PET/CT pathology at EOT (Fig. 2C). Within group 3, BQ-treated lesions were overrepresented in cluster 3a, which was characterized by severe PET/CT pathology that demonstrated marked improvement at EOT, and underrepresented in clusters 3b and 3c, which were characterized by much less improvement in PET/CT pathology (result not statistically significant). These data suggest that BQ improved pathology in lesions with severe PET/CT pathology at the start of treatment more effectively than other treatment arms, such that fewer lesions with severe PET/CT pathology remained at EOT. This effect was specific to bedaquiline and quabodepistat in combination; although DQ- and DBQ-treated lesions were slightly overrepresented in the same clusters 2a and 3a that showed improvement in lesions with severe PET/CT pathology, the differences from the baseline were not statistically significant (Fig. 2C). DBQ-treated animals had significantly fewer (adj.res <−1.96) lesions in cluster 3b (characterized by high pathology and high bacterial burden) than other drug treatments, indicating that like the other higher-order combinations, DBQ more effectively reduced bacterial burden in lesions with severe PET/CT pathology, such that fewer of these high burden, high pathology lesions remained at EOT. Taken together, these data suggested that quabodepistat in combination with bedaquiline improved pathology measurable by PET/CT more than other treatments. The effect was specific to combination with bedaquiline and could be diminished with additional drugs.

### Multivariate profiles describe lesion-specific responses to treatment during subsequent treatment

Serial PET/CT scans enabled us to divide the 8 weeks of treatment time (complete treatment) into phases: we called the first four weeks “initial treatment” and the second four weeks “subsequent treatment.” Improvements in signs of disease in the animals by four weeks suggested that the midpoint of treatment would be suitably information-rich for analysis. Based on the alignment of marmoset responses during complete treatment to clinical treatment outcomes, we hypothesized that similar analyses of subsequent treatment in marmosets would be informative of clinical treatment phase differences. We compiled a second set of multivariate profiles that included per-lesion PET/CT measures from the end of subsequent treatment (i.e., EOT, after eight total weeks of treatment), changes in PET/CT pathology during subsequent treatment (between four and eight weeks of treatment), and the bacterial burden at EOT. These profiles included an additional PET feature (12 total), the change in total lesion glycolysis during subsequent treatment, whose parallel feature from the complete treatment analysis was collinear with other features and, therefore, removed for the complete treatment analysis. We aimed to categorize groups of lesions with similar feature profiles to identify unique patterns that described how lesions evolve and respond to drug treatment during subsequent treatment. We used unsupervised clustering to identify nine distinct lesion populations based on similar response profiles during subsequent treatment (Fig. S11, S12). We performed parallel clustering analyses in which the subsequent treatment period was defined by alternate time points (i.e., not the midpoint): in the first, the periods were divided into 0–2 weeks initial/2–8 weeks subsequent treatment, and in the second, the periods were divided into 0–6 weeks initial/6–8 weeks subsequent treatment.

**Figure 3.**
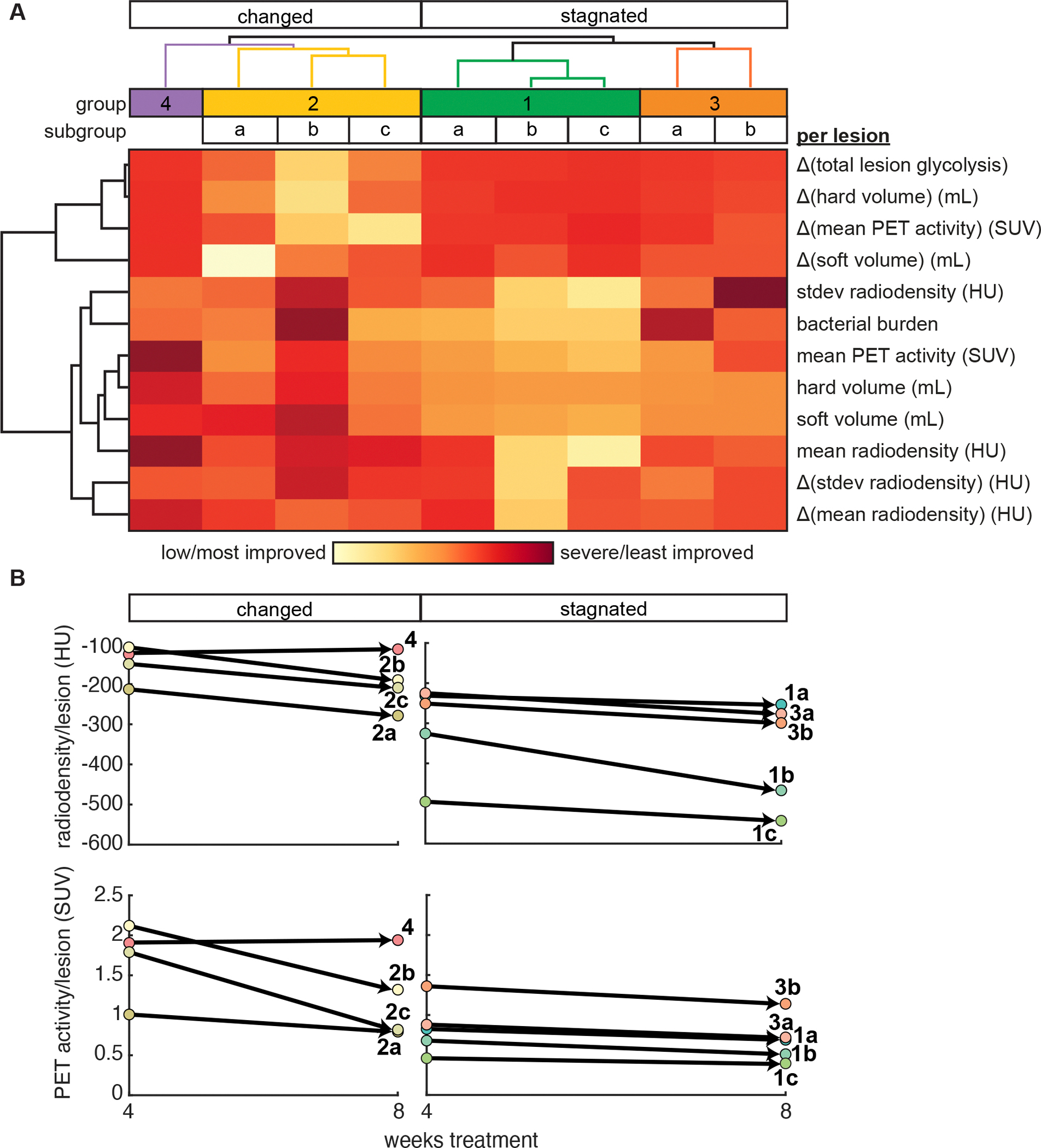
Characteristics of each lesion cluster from subsequent treatment phase analysis and cluster distribution across all lesions. **(A)** Heatmap of hierarchical clustering of median feature values across the clusters from Leiden community detection. Clusters are split into two large branches: changed and stagnated. Four groups (1, 2, 3, 4) of similar clusters are labeled from the hierarchical clustering, and clusters are labeled (a, b, c) within each group. Feature values were scaled for visualization. The severe/low color scale is relative to the values in this visualization (not absolute). Δ refers to changes in features from the middle (4 weeks) to end of treatment (EOT, 8 weeks). **(B)** Median values for mean radiodensity per lesion (CT measure, top) and mean FDG activity per lesion (PET measure, bottom) at the middle (4 weeks) and end (8 weeks) of treatment for each cluster (labeled # for group and a-c for cluster).

To compare the feature profiles that defined each subsequent treatment phase cluster of lesions, we evaluated the median feature values of lesions within each cluster and performed hierarchical clustering on the aggregate features (Fig. 3A, Table S3). The feature profiles were split into two large branches and four groups across those branches (two groups per branch, ordered 1–4 in order of increasing severity). The large branches were distinguished by changes in or stagnation of PET and some CT features (hard and soft volumes) during subsequent treatment. The groups within each branch had broad commonalities in PET/CT features at the end of drug treatment and specific feature differences that distinguished their member clusters. Lesions from clusters in group 1, which was a member of the branch of lesion profiles that did not show much change in PET/CT features during subsequent treatment, were characterized by the lowest PET/CT pathology features and low bacterial burden. The lack of improvement in PET/CT pathology during subsequent treatment in these lesions suggested that any improvements to their pathology occurred during initial treatment (Fig. 3B, fig. S13). Lesions from clusters in group 2 are characterized by moderate or severe PET/CT pathology features that improved (i.e., decreased) during subsequent treatment. Bacterial burden was uniformly low in lesions from cluster 2c but varied widely in clusters 2a and 2b. Lesions from clusters in group 3 were characterized by moderate or severe PET/CT pathology, features that stagnated (did not improve nor worsen) during subsequent treatment. Bacterial burden was uniformly high in lesions from cluster 3a but varied widely in cluster 3b. Lesions from the group 4 cluster were characterized by severe PET/CT pathology, features that worsened during subsequent treatment. Bacterial burden varied widely in cluster 4c, which suggested that worsening lesion pathology was not coupled with high bacterial burden. The parallel clustering analyses of different time points did not offer new information that was not already discerned from the complete treatment analysis or the midpoint-based analysis (Fig. S14). Therefore, we focused our additional analyses on the subsequent treatment clusters defined using the midpoint of treatment.

**Figure 4.**
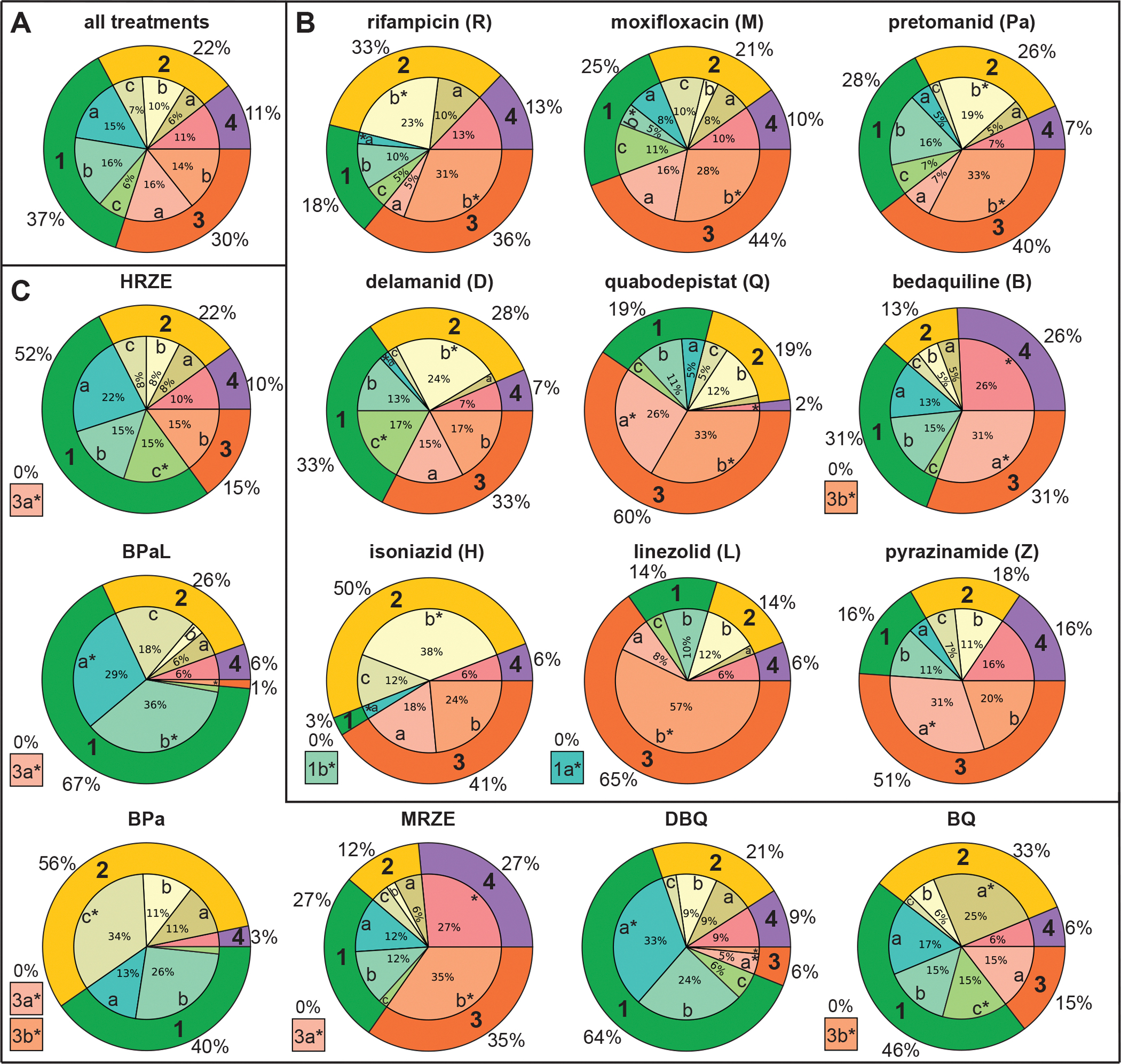
Distribution of subsequent treatment phase clusters across lesions from individual treatment arms. Inner pie charts represent the analysis of distribution of the clusters from the subsequent treatment phase (second half of treatment). Cluster groups are indicated in the outer pie charts: (1) low bacterial burden and low PET/CT pathology with minimal improvement during subsequent treatment; (2) varied bacterial burden and varied improvement in PET/CT pathology during subsequent treatment; (3) varied or high bacterial burden and severe PET/CT pathology with minimal improvement during subsequent treatment; (4) varied bacterial burden and severe PET/CT pathology that did not improve or worsened during subsequent treatment. Outer wedge colors correspond to those in Figure 3. *Indicates significant deviation (95% confidence interval using significance threshold of 1.96) from the expected (average) number of lesions in a specific cluster by adjusted residual analysis following the chi-squared test for homogeneity (p < 0.001). **(A)** Distribution of the clusters across all lesions from all drug treatments, representing the average distribution across drug treatments (n = 1193 lesions). **(B)** Distribution of the clusters across all lesions for each of the monotherapy treatment arms. Monotherapies are ordered by ranking from total lung CFU (Figure S1A). **(C)** Distribution of the clusters across all lesions for specific combination treatment arms. Boxes to the left of each pie chart in panels B and C indicate significant underrepresentation from specified clusters that are completely absent. Unlabeled inner slices are <5% of the total for that drug treatment. The distributions of clusters across the remaining seven treatment arms not explicitly discussed are shown in Fig. S15.

The distribution of lesions from all treatment arms across the subsequent treatment phase clusters represented the baseline to compare individual drug treatments to all treatments (Fig. 4A). 67% of all lesions stagnated after initial treatment (groups 1 and 3 combined), whereas the other 33% (groups 2 and 4) changed during subsequent treatment (some for better, some for worse). Within those two branches, 11% of the total lesions were represented in the group with worsened pathology (group 4, purple) during subsequent treatment, 22% were represented in the group with improving pathology (group 2, yellow), 37% stagnated with low pathology (group 1, green), and 30% stagnated with moderate or severe pathology (group 3, orange). Because our analysis of complete treatment outcomes indicated that deviations from the baseline distribution can be used to interpret treatment outcomes, we applied the same principle to the subsequent treatment phase clusters. We hypothesized that deviations from the baseline could be used to identify drug treatments that resulted in a greater abundance of improving lesions (and thus, fewer worsening lesions or fewer lesions that stagnated with moderate or severe pathology). We evaluated the distribution of subsequent treatment phase clusters across all lesions in each of the monotherapy treatment arms (Fig. 4B). Across all lesions within each of the nine monotherapy treatment arms, lesions that stagnated with severe pathology or lesions with worsened pathology were significantly overabundant (adj.res > 2.58) in seven out of nine arms, and lesions that continued to improve or stagnated with low pathology after initial treatment were underrepresented (adjusted residual analysis after chi-squared test for homogeneity, significance thresholds ±1.96 and ±2.58 corresponding to 95% and 99% confidence intervals, respectively). We demonstrated in the complete treatment analysis that monotherapies failed to adequately target lesions that presented with severe PET/CT pathology at the start of treatment, resulting in an abundance of lesions with high pathology and high bacterial burden remaining at EOT (Fig. 2B). Taken together with the subsequent treatment phase analysis, the data suggested that this failure could be attributed to the stagnation in improvement that occurs after initial treatment. The two exceptions to this overabundance of stagnation were delamanid and isoniazid. Isoniazid-treated lesions were significantly overrepresented (adj.res > 2.58) in cluster 2b, characterized by continued improvement in PET/CT pathology and high bacterial burden, and underrepresented in the clusters of group 1, which stagnated with low pathology. These data suggested that the strength of isoniazid in subsequent treatment was its continued slow-but-steady targeting of lesions with severe pathology. Delamanid had significant overrepresentation (adj.res > 2.58) in cluster 2b but similar representation across the broad groups (1–4) to all treatment arms, suggesting potential continuing activity in later treatment that could be explored in future studies.

### The efficacy of HRZE is limited to initial treatment

We next evaluated the distribution of subsequent treatment phase clusters across all lesions in animals treated with HRZE (Fig 4C). HRZE-treated lesions were significantly overrepresented (adj.res > 1.96) in cluster 1c, which was characterized by stagnation with low PET/CT pathology after initial treatment, and underrepresented (adj.res <−2.58) in cluster 3a, which stagnated with severe pathology and high bacterial burden. These deviations from other treatments suggested that after initial treatment, low/moderate pathology lesions treated with HRZE were more likely to resolve than with other treatments, and severe pathology lesions were less likely to remain with high bacterial burden. However, the total proportion of HRZE-treated lesions that stagnated after initial treatment (groups 1 and 3 combined) was 67%, the same as the overall proportion of lesions that stagnated across all treatment arms. Furthermore, the proportion of lesions that worsened during subsequent treatment with HRZE was not different from the baseline. These data suggested that although HRZE effectively targeted the bacterial burden in severe pathology high-burden lesions, most of its activity occurred during initial treatment, and it did not induce more improvement or prevent more worsening in pathology during subsequent treatment than other treatment arms in this study.

### Greater efficacy of BPaL during subsequent treatment

We next evaluated the distribution of lesions across subsequent treatment phase clusters in animals treated with BPaL (Fig. 4C). BPaL-treated lesions were significantly overrepresented (adj.res > 2.58) in clusters 1a and 1b, which stagnated after initial treatment with low PET/CT pathology, and in cluster 2c (adj.res > 2.58), which was characterized by low bacterial burden, moderate PET/CT pathology, and marked improvement (decrease) in PET activity. BPaL-treated lesions were significantly underrepresented (3a adj.res < −1.96 and 3b adj.res < −2.58) in group 3 clusters, which stagnated with severe pathology, and underrepresented in cluster 4 (not statistically significant). The combined overrepresentation of low pathology and improving lesions and underrepresentation of lesions that stagnated with severe pathology or worsening pathology suggested that BPaL was effective during both phases of treatment: the low and moderate pathology, “easier-to-treat” lesions were resolved during initial treatment (falling in group 1), and the lesions that started with moderate or severe pathology were continuously targeted after initial treatment, resulting in fewer severe lesions that stagnated (group 3) and an abundance of lesions that showed continued improvement after subsequent treatment (group 2). These findings suggest another mode by which BPaL outperforms HRZE that could not have been determined with rank by single outcome measures.

The value of adding linezolid to BPa has been debated; Mtb strain-specific antagonism has been reported and linezolid’s toxicity reduces patient compliance (20). The distribution of BPa-treated lesions from the complete treatment analysis was comparable to HRZE-treated lesions (Fig. S10). To understand the additional effects of linezolid in combination with BPa during subsequent treatment, we evaluated the distribution of BPa-treated lesions across the subsequent treatment phase clusters (Fig. 4C). Unlike BPaL-treated lesions, BPa-treated lesions were not significantly over- (or under-) represented in group 1 (which stagnated with low pathology). However, BPa-treated lesions were significantly overrepresented (adj.res > 2.58) in cluster 2c (characterized by improvement/decrease in PET activity) and significantly underrepresented (adj. res <−2.58, specifically, they were completely absent) from group 3 (those lesions that stagnated with moderate or severe PET/CT pathology). BPa-treated lesions were also underrepresented in cluster 4c, more so than BPaL, though this result did not reach statistical significance. The distribution of BPa-lesions across the clusters suggested that, like BPaL, BPa is effective during both phases of treatment: low and moderate pathology lesions were resolved during initial treatment (falling in group 1). However, BPa was distinguished from BPaL in its slightly greater activity against lesions with moderate to severe pathology during subsequent treatment; lesions that might have stagnated with moderate or severe pathology during subsequent treatment with BPaL (group 3) were targeted by BPa, such that they continued to improve (group 2), and fewer lesions worsened.

### MRZE-treated lesions get worse during subsequent treatment

We demonstrated in the analysis of marmoset response to complete treatment (two months) with MRZE that its reduction in bacterial burden was not coupled with improvement in PET/CT pathology (Fig. 2C). To investigate the effects of MRZE during subsequent treatment, we evaluated the distribution of subsequent treatment phase clusters across all lesions in animals treated with MRZE. Strikingly, MRZE-treated lesions were significantly overrepresented (adj.res > 2.58) in cluster 4, which was characterized by severe PET/CT pathology that worsened during subsequent treatment (Fig. 4C). MRZE-treated lesions were also significantly overrepresented (adj.res > 2.58) in cluster 3b, which stagnated with severe pathology after initial treatment. The underrepresentation of MRZE-treated lesions from lesion cluster 3a, which stagnated with severe pathology and had high bacterial burden, was statistically significant (adj.res < −2.58) but unsurprising; treatment with MRZE was shown to reduce bacterial burden similarly to the other higher-order treatment arms (Fig. S1A). The majority of MRZE-treated lesions (62%) stagnated after initial treatment (groups 1 and 3 combined), and only 12% continued to improve during subsequent treatment (group 2). Taken together with the previous analysis (Fig. 2), these data suggested that although the total lung bacterial burden in MRZE-treated animals was statistically indistinguishable from HRZE or BPaL, the reduction of bacterial burden in MRZE-treated lesions did not coincide with a decrease in pathology during the same time frame as it did for HRZE and BPaL. More than 50% of HRZE- and BPaL-treated lesions fell in group 1 (“easier-to-treat” lesions that were targeted during initial treatment). In contrast, only 27% of MRZE-treated lesions fell in group 1 and 35% in group 3, which stagnated with severe pathology after initial treatment, suggesting that HRZE and BPaL both had greater abilities to simultaneously reduce pathology along with bacterial burden during initial treatment. Furthermore, MRZE had negative utility during subsequent treatment; more lesions with severe PET/CT pathology worsened with MRZE treatment than with other drug treatments.

### Quabodepistat is more active during initial treatment

We demonstrated that two months of treatment with quabodepistat combined with bedaquiline resulted in more PET/CT pathology improvement (overrepresentation of the greatly-improved cluster 2a lesions) than other treatment arms (Fig. 2C). To investigate whether this effect was limited to initial treatment or continued beyond initial treatment, we evaluated the distribution of subsequent treatment phase clusters across all lesions in animals treated with DBQ and BQ. DBQ-treated lesions were significantly overrepresented (adj.res > 2.58) in cluster 1a (which stagnated after initial treatment with low PET/CT pathology) and significantly underrepresented (adj.res < −1.96) in group 3 clusters (which stagnated after initial treatment with moderate or severe PET/CT pathology). 70% of all DBQ-treated lesions fell in groups 1 or 3 (the groups whose PET/CT pathology stagnated), and only 21% continued to improve (group 2), which was not different from the baseline distribution of all treatments (Fig. 4C). These proportions suggest that most of DBQ’s activity quantifiable by PET/CT pathology was limited to the initial treatment phase.

BQ-treated lesions were significantly overrepresented (adj.res > 1.96) in cluster 1c (stagnated after initial treatment with low PET/CT pathology) and in cluster 2a (marked by a decrease in soft volume during subsequent treatment, adj.res > 2.58) and were significantly underrepresented (adj.res < −2.58) in cluster 3b (stagnated with severe pathology) (Fig. 4C). 61% of all BQ-treated lesions fell in groups 1 and 3, and 33% continued to improve (group 2). These proportions suggest that though BQ continued to target lesions with moderate to severe pathology more effectively than DBQ after initial treatment, the majority of improvement observed in the complete treatment analysis of BQ-treated animals occurred during initial treatment, such that the majority of lesions stagnated in their improvement of pathology during subsequent treatment. Taken together with the complete treatment analysis, our data suggested that these quabodepistat regimens were not superior to the current standard of care.

### Combining histopathological classification with clustering distinguishes responding and non-responding cavitary granulomas

Cavitary disease is considered a more severe manifestation of human pulmonary tuberculosis and is associated with a higher risk of treatment failure and relapse (21). We hypothesized that lesions with more severe PET/CT pathology features represent severely diseased lesions and, therefore, these lesions would not resolve within the initial treatment timeframe and would require continued treatment to resolve (the four-week subsequent treatment phase or even longer). We inspected the lesion clusters that stagnated with low PET/CT pathology features during subsequent treatment to determine if any of these lesions presented with severe pathology at the start of treatment that decreased rapidly to low pathology within the first four weeks of treatment. We observed that no cluster of lesions with severe PET/CT pathology at the start of treatment (clusters from groups 2, 3, and 4) displayed rapid, direct improvement to low PET/CT pathology within the first four weeks of treatment (Fig. S16). The lesions that stagnated with low pathology after initial treatment (from the clusters in group 1) presented with low to moderate pathology at the start of treatment. Lesions that improved (decreased) in PET/CT pathology during subsequent treatment (group 2) had more severe pathology at the start of treatment. These data suggested that lesions with more severe PET/CT pathology required longer treatment time to resolve, which supports the clinical treatment decision to lengthen the treatment duration (beyond 6 months) for patients with cavitary (i.e., more severe) disease when that information is available.

**Figure 5.**
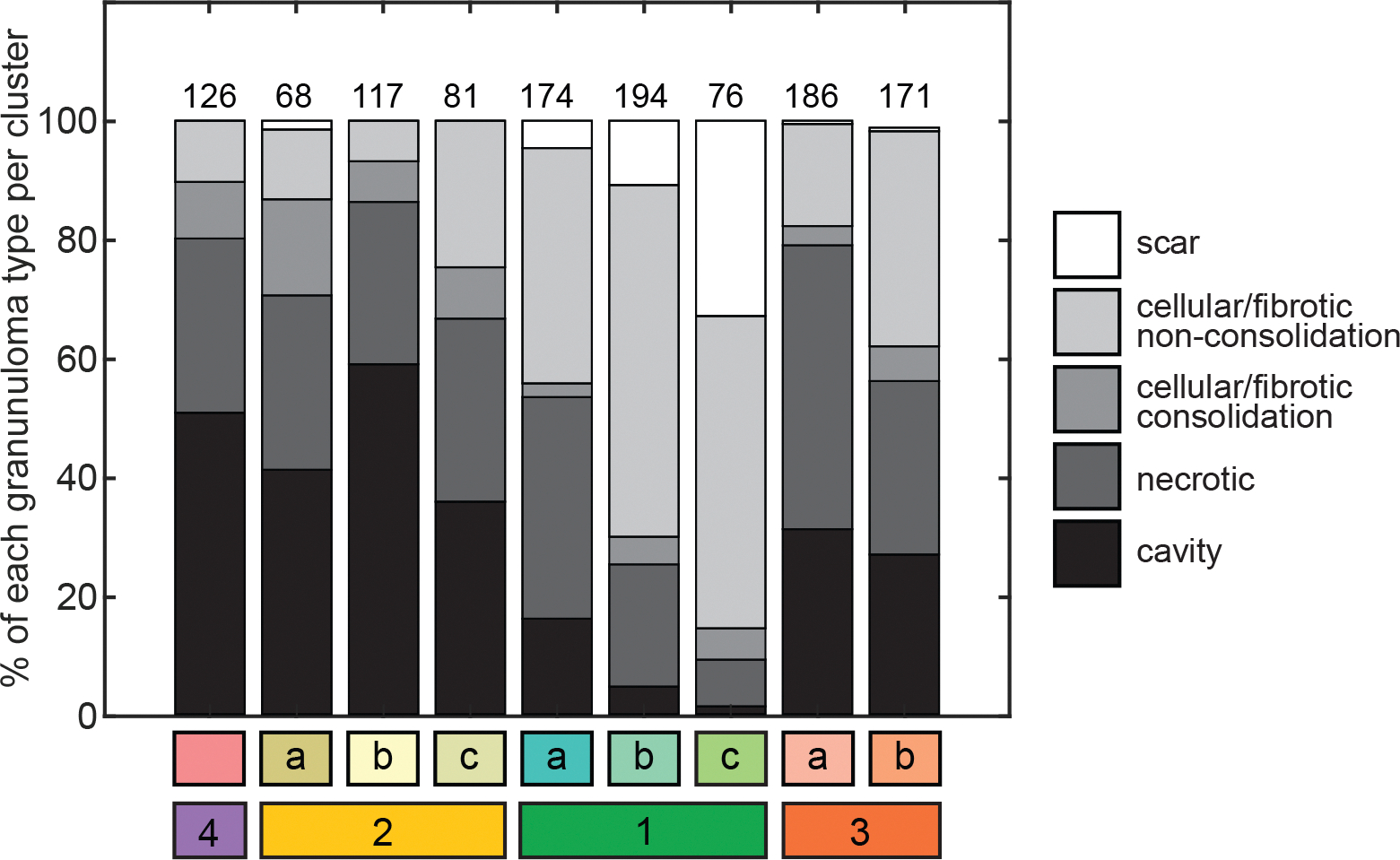
Distribution of lung granuloma types across the subsequent treatment phase clusters. Each stacked bar represents the percentages of each of the granuloma types present in each cluster from the subsequent treatment phase analysis. Cluster colors correspond to those defined in Figure 3. The number above each bar is the total number of lesions in that cluster.

Because the presence of cavitation in human disease is a criterion for extended treatment duration, we asked if the lesions that were “most difficult to treat” in the marmoset - in that they worsened or stagnated with severe pathology during subsequent treatment - were cavitary granulomas. Each lesion was labeled with its histopathological granuloma classification (cavitary, necrotic, cellular/fibrotic consolidation, cellular/fibrotic non-consolidation, or scar) at necropsy and matched to its corresponding lesion on PET/CT scans. Across all (1,193) lesions, 332 (28%) were cavitary, 364 (31%) were necrotic, 71 (6%) were cellular/fibrotic consolidation, 367 (31%) were cellular/fibrotic non-consolidation, and 59 (5%) were scars or resolved lesions. We evaluated the distribution of granuloma types across the subsequent treatment phase clusters. We observed that the majority (51%) of lesions in cluster 4 (the lesions with worsened PET/CT pathology) were cavitary granulomas at necropsy, 29% were necrotic granulomas, and 21% were cellular/fibrotic (Fig. 5). We posited that these cavitary granulomas were not successfully targeted or were unresponsive to drug treatment. 59% of the granulomas in cluster 2b, which was characterized by severe but improving PET/CT pathology and high bacterial burden, were also cavitary (Fig. 5). The changes in PET/CT features enable distinction between lesions from these two clusters; whereas the cavitary granulomas of cluster 4 were not responding to treatment, the cavitary granulomas of cluster 2b were improving, and we hypothesize that they would have continued to improve with longer treatment time. Taken together, we have shown that quantitative PET/CT measures can be used to assess tuberculosis treatment outcomes at the lesion level and that a combination of these features is more informative of response to treatment than any single metric alone.

## DISCUSSION

Biomarkers that can reliably predict non-relapsing cure for pulmonary tuberculosis are necessary to choose the best treatment candidates for clinical trials. The critical challenge is that bacterial burden and sputum conversion, well-established study endpoints, are not always correlated with non-relapsing cure (8). PET/CT imaging markers showed promising correlations with relapse outcomes in patients with both drug-susceptible and multi-drug resistant TB, but these markers have not been fully explored in non-human primates (NHPs) for their potential to predict treatment duration and relapse for drug regimen development and optimization (10, 11). We hypothesized that longitudinal radiological studies in marmosets, which develop advanced cavitary disease, combined with endpoint bacterial burden, could inform clinical outcomes for cavitary tuberculosis better than bacterial burden data alone. We determined that individual PET/CT markers were uncorrelated and therefore asked if lesion-specific PET/CT markers combined with bacterial burden would inform what we have seen in clinical outcomes in hindsight. We found that multivariate profiles of lesion-specific features do align with known clinical treatment outcomes, establishing the utility of PET/CT imaging in the marmoset model for the prediction of non-relapsing cure for new drug regimens to treat cavitary tuberculosis.

Estimates of clinical relapse rates after completion of the 6-month HRZE treatment regimen range from 3% to 15% (7, 22, 23). In our study, we observed that some animals treated with HRZE, but not all, had lesions that failed to resolve pathology detectable by PET/CT, despite the reduction of bacterial burden, suggesting that this phenomenon varied from animal to animal. This animal-to-animal variability echoes clinical variability and could explain the variability seen in clinical relapse rates. It may be that a subset of patients with drug-susceptible TB sputum convert and retain some low-burden lesions with severe pathology, similar to our observation in the marmoset. The severe pathology of these lesions provides a more favorable environment for the few remaining bacilli to tolerate the duration of drug treatment and resume growth upon cessation of treatment (or some time after), allowing for relapse. The lack of association between bacterial burden and pathology in these high-pathology lesions is striking and suggests a differential impact of drug combinations on the immune response. Future marmoset studies that incorporate immune biomarker detection (e.g., immunofluorescent staining and flow cytometry analysis of granuloma cells (24) and mass spectrometry-based multiplexed imaging) may identify host-directed effects of combinations or targets for host-directed therapies.

The approval of BPaL for MDR-TB was a critical success for TB regimen development. This regimen substantially shortened the treatment time for MDR-TB and extensively drug-resistant (XDR) TB (25). Though preclinical studies in mouse models have demonstrated BPaL’s superiority to HRZE, BPaL and HRZE have not been directly compared in clinical trials (20). Our results from lesion-specific, treatment-specific analysis in NHPs indicate that while the reductions in bacterial burden by HRZE and BPaL are comparable, the reduction in lesion pathology differs. BPaL treatment improved marmoset lesion pathology, in contrast to HRZE treatment. Furthermore, BPaL treatment resulted in continued activity during subsequent treatment, whereas HRZE treatment did not. Therefore, our analysis of NHP lesions suggests that BPaL may be superior to HRZE in the reduction of clinical relapse rates for the treatment of drug-susceptible TB. The front-line agents in this study have been reported in clinical PET/CT trials (26); formal comparison of these responses across humans and marmosets may provide critical insight to establish the direct predictive value of marmoset responses to drug treatment.

Fluoroquinolones have demonstrated potent antimycobacterial activity against drug-resistant strains (27, 28). Early bactericidal activity (EBA) studies of fluoroquinolones have shown comparable activity to isoniazid, indicative of the capability of rapid clearance (29, 30). Preclinical animal studies for combinations that included fluoroquinolones showed earlier culture conversion than HRZE and, therefore, were thought to hold great promise for treatment shortening (31). However, the REMox TB trial (and other similar trials), which tested the treatment-shortening potential of MRZE (4 months compared to the 6 months with HRZE), did not establish non-inferiority, raising the question of the reliability and predictive value of preclinical models (9). Patient-level analysis of data from these trials distinguished populations in which the four-month regimens were non-inferior, namely individuals with <2+ sputum smear grades or non-cavitary disease (32). The four-month regimen was found to be inferior to the six-month regimen in individuals with 3+ sputum smear and cavitary disease. Our multivariate modeling of treatment outcomes in marmosets indicates that whereas bacterial burden is reduced in MRZE-treated animals comparably to HRZE and BPaL, pathology is not reduced in the same time frame, and worsens beyond the first four weeks of treatment. MRZE-treated animals retain a proportion of lesions with low bacterial burden and severe pathology that may provide a protective niche for potential relapse. Our results indicate that PET/CT data in the marmoset model could have predicted the failure of MRZE in clinical trials and that failure is specifically associated with the failure to adequately target cavitary lesions and reduce lesion pathology. Though there is no clinical evidence of inferiority of MRZE to HRZE when both regimens are given for six months, the results of our marmoset study suggest that MRZE may perform worse than HRZE even if given for six months. Results from our subsequent treatment analysis indicate that more severe lesions require longer treatment time, and MRZE demonstrated inadequacy against those lesions. Our finding that MRZE successfully targets the lower pathology lesions (i.e., the lesions in group 1 from the complete treatment analysis, Fig. 2 and group 1 from the subsequent treatment analysis, Fig. 4) aligns with the non-inferiority result in patients with <2+ sputum grades and non-cavitary disease. Therefore, our study elucidates a lesion-level explanation of MRZE’s failure after two months of treatment in the marmoset that is agnostic to the difference in total treatment times in the human clinical trials.

Preclinical models typically focus on a single regimen throughout the entire duration of therapy rather than regimen cycling. We demonstrate in this study that different drug treatments vary in effectiveness over time, supporting regimen cycling during the course of treatment. HRZE’s utility in the reduction of pathology was limited to initial treatment, whereas pathology was effectively reduced throughout the entire eight-week course of treatment with BPaL and BPa. Quabodepistat treatment resulted in a marked reduction in lesion pathology, but this effect was largely limited to initial treatment. Our data suggest that higher-order multi-drug treatment may not be necessary to achieve a reduction of pathology during the later stages of treatment. Conflicting outcomes regarding the superiority of BPaL to BPa have introduced some doubts about the strengths of the BPaL regimen (20). In this study, BPa treatment resulted in more improvement and less stagnation of lesion pathology detectable by PET/CT compared to BPaL during subsequent treatment. Considering the understandable challenge of medication compliance with linezolid due to toxicity, and based on our subsequent treatment phase analysis, we suggest that discontinuing linezolid after an appropriate initial treatment phase with BPaL may be of benefit to patients. Our results also indicate a use for real-time PET/CT imaging to optimize the two phases of drug treatment during regimen development. To use this platform to study regimen cycling, future studies could be designed to compare 8 weeks of a constant treatment (e.g., BPaL) to 4 weeks of the same treatment plus another 4 weeks with a step-down or other change to the combination (e.g., BPaL/BPa).

We observed that most lesions that worsened during subsequent treatment and exhibited severe pathology at the end of treatment despite marked, continuous improvement were determined to be cavitary at necropsy. Our analysis enabled the separation of unresponsive cavitary lesions to treatment from cavitary lesions that responded and improved with treatment (but did not resolve within two months). Based on the continuous improvement of the latter group of cavitary lesions, we hypothesize that those lesions would continue to resolve with longer treatment time. The extended duration of cavitary lesion response to treatment supports the presence of cavitary disease on initial X-ray as a criterion for longer treatment time, though we acknowledge that most TB patients do not have access to X-rays. The identification of non-responding cavitary granulomas highlights the importance of designing and using treatments that successfully target cavitary granulomas. Taken together, our study demonstrates the utility of PET/CT to identify lesion-specific, treatment-induced changes indicative of treatment success or failure. Indeed, in the clinical companion study, treatment-recalcitrant cavitary lesions were identified using PET/CT to be responsible for disease relapse in human patients. This highlights the importance of preclinical models and methods that accurately recapitulate the pathology and responses seen in human TB.

Our study has some critical limitations. This study has no untreated control group because the untreated animals would not live past eight weeks of infection to enable comparison against the treated animals. Therefore, we can only compare treatments against the aggregate behavior of all treatments in the study. It is possible that the clustering could change with additional input data if more animals or drug treatments were tested. However, the results of the leave-one-out analysis support the possibility of similarly interpretable results with the addition of new drug regimens to this dataset. Because we demonstrated that the patterns in the dataset are stable and not dependent on any particular treatment arm of the 22 in this study, tuning the clustering resolution with the addition of new drug regimens should result in interpretable results and prediction of clinical outcomes. Currently, our study does not inform in-class comparisons; such comparisons could be more informative as new treatment arms are added. Future studies can also be expanded to evaluate drug-specific effects in monotherapies versus combinations and to include combinations that are treatment-shortening in clinical trials. Our drug regimen set, for example, does not include the 4-month isoniazid-rifapentine-pyrazinamide-moxifloxacin (HRpZM) regimen, which has demonstrated treatment-shortening potential compared to HRZE. Additionally, the timeframe of the experiments in this study (2 months) is shorter than the human course of treatment (minimum 4–6 months). Nevertheless, the results after two months of treatment are interpretable and align with clinical efficacy outcomes. Future studies may be conducted to explore lesion resolution rates and patterns with regimens that have been shown to be treatment-shortening, which could inform future strategies for real-time prediction of treatment efficacy and shortening. Another limitation of the study is that the marmoset may not model all aspects of severe TB disease and, therefore, outcomes in patients with very difficult-to-treat disease may not be well aligned with those in the marmoset.

FDG-PET/CT is expensive and is not logistically feasible globally, which may limit these studies to regimen development and exploration. Therefore, this type of study may not be feasible for phase III clinical trials. Despite these limitations, however, our study establishes the utility of two tools for drug regimen studies: (1) the marmoset is a valuable model of TB infection that allows for lesion-specific study and responds to drug treatment in a manner that aligns with clinical outcomes, (2) FDG-PET/CT imaging allows for real-time analysis of evolution of lesion pathology in response to drug treatment for the evaluation of treatment success and treatment phase development. Because the analysis is focused on lesion evolution and not exclusively bactericidal activity, it is agnostic to lesion age and bacterial strain. Application of this analysis strategy to human clinical PET/CT data promises to address a critical need for improving our ability to identify the best combinations to achieve durable cure in all TB patients.

## MATERIALS AND METHODS

### Animals, ethics assurance, and pharmacokinetics.

All procedures with *Callithrix jacchus* (common marmosets), including breeding in NIH facilities, were in accordance with the recommendations of the Guide for the Care and Use of Laboratory Animals of the National Institutes of Health and approved by the NIAID Animal Care and Use Committee in Protocol LCIM-9 (Permit issued to NIH as A-4149–01). Both male and female marmosets ages 2 to 6 years old were included in the study but as group numbers were small, no sex-specific analyses were conducted. Efforts were made to minimize suffering and provide intellectual and physical enrichment as approved in LCIM-9. Agent pharmacokinetic parameters and tolerability of chosen doses were determined in naïve adult marmosets in BSL-2 housing as previously described (13, 33). Additionally, steady-state pharmacokinetics were determined during tolerability studies in naïve animals and during treatment for the Mtb infection commencing after 3 weeks of treatment (Table S4).

### Study design

Common marmosets (*Callithrix jacchus*) were infected with *M. tuberculosis* H37Rv via aerosol delivery as previously described (13, 14). A baseline PET/CT scan was taken one month post-infection. Animals were randomly assigned to 22 treatment arms at 6–7 weeks post-infection and started daily oral treatment. Baseline lesion volumes across each arm were evaluated to ensure balanced disease burden across treatment arms (Table S1, Fig. S17). PET/CT scans were taken at the start of treatment and every two weeks thereafter until eight weeks of treatment, after which the animals were humanely sacrificed. A PET/CT image-guided necropsy was performed for each animal, with organ weights and samples of each lesion collected for bacterial enumeration and histopathological classification. PET/CT and bacterial burden per-lesion features were combined in unsupervised clustering to compile multivariate profiles to describe lesion-specific responses to treatment.

### Infection, infection monitoring, and lesion collection.

Prior to infection, marmosets were transferred to a BSL-3 animal facility approved for the containment of Mtb, housed in pairs, and handled in sets of 6 to 8 animals as previously described (13, 14). All 112 Marmosets were infected with the same stock of Mtb H37Rv with a nose-only aerosol generated by a BANG nebulizer through a CH Technologies inhalation system (Westwood, NJ) that generated 10–25 granulomas per animal assessed after 4 weeks of infection by FDG-PET/CT. Marmosets were treated orally once a day (7 days/week) with the drugs and combinations of drugs listed in Table 1 (and Table S4) for 2 months beginning 6–7 weeks after infection when the animals were observed to have multiple lesions in their lung on the pre-treatment PET/CT and weight loss had commenced (13, 33). The treatments were distributed so that each treatment was administered during 2 to 3 infection sets until 5 animals had been treated with each agent or regimen. During treatment, each animal was monitored by FDG-PET/CT after 2, 4, 6, and 8 weeks to confirm that the marmoset was responding to treatment and to measure the changes in lesion volume, density, and FDG uptake. A PET/CT guided necropsy plan was made for each animal so that major pulmonary lesions, including cavities, could be located and collected for histologic assessment and bacterial enumeration in triplicate onto Middlebrook 7H11 agar plates containing albumin, oleic acid, dextrose, and 0.4% w/v charcoal as previously described (33). Development of resistance during treatment was assessed either by picking 5–10 of the resulting colonies per lesion and determining the minimum inhibitory concentration (MIC) for the agent(s) the lesions had been exposed to or by plating diluted lesion homogenates onto M7H11 agar containing 5X and 20X MIC concentrations of the agent(s) and looking for colony growth in comparison to M7H11 agar without the agents. Phenotypic resistance to Pa was detected in two animals by both methods above.

### Computational analysis

Two animals and one isolated lesion from a third animal developed repeated phenotypic resistance; these animals/lesions were removed from subsequent analysis (noted in Table 1). Prior to clustering, features were checked for redundancy using Pearson correlations across all pairs. Features with a correlation coefficient >0.75 or <−0.75 were considered redundant and removed from subsequent analysis. Lesions without valid colony-forming unit (CFU) counts were removed. Using the remaining features (11 for complete treatment analysis, 12 for subsequent treatment phase analysis), data were normalized and scaled using the standard scaler from Python *sklearn*. Data were linearly transformed using principal component analysis (PCA), and all components were used to build a nearest neighbors graph (using cosine distance metric). Leiden community detection (LCD) was used to detect dense communities in the neighbors graphs for lesion clustering using the Python package *scanpy*. Clustering results were robust to multiple algorithms, including Louvain community detection (*scanpy*) and agglomerative clustering (*sklearn*) (Fig S8). Resolution for LCD was 0.6 for the complete treatment analysis and 0.7 for the subsequent treatment phase clustering. Hierarchical clustering was performed using the clustergram function with complete linkage and cosine distance from Python package *dashbio*.

### Statistical analysis

To determine whether the distribution of cluster assignments (i.e., what cluster each lesion was categorized in) across the drug treatments was the same, we used the chi-square test for homogeneity. Rejection of the null hypothesis (p<0.05) suggested that the distribution of observations across clusters is not the same for all drug treatment arms. Because the chi-square test for homogeneity indicated (p<0.001) that there were significant differences across the drug treatment arms, we used the adjusted residual analysis post-hoc test to identify which specific drug treatments differed from the expected (baseline) for each cluster using a significance threshold of ±1.96 for a 95% confidence interval and ±2.58 for a 99% confidence interval. A positive adjusted residual indicated that the observed count of lesions in a given cluster was higher than expected, and a negative adjusted residual indicated that the observed count was lower than expected.

## Supplementary Material

Supplemental Materials

MDAR Reproducibility Checklist

List of Supplementary Materials:

Materials and Methods

Fig. S1 to S20

Tables S1 to S5

MDAR

## Figures and Tables

**Table 1.** Study drug treatment arms and number of marmosets per arm.

Monotherapies (abbreviation, # animals)	Pairwise drug combinations (# animals)	Higher-order drug combinations (# animals)

bedaquiline (B, 5)	BD (5)	BPaL (5)
delamanid (D, 6)	BL (5)	DBQ (5)
isoniazid (H, 4)	BQ (5)	HRZE (5)
linezolid (L, 5)	BPa (5)	MRZE (5)
moxifloxacin (M, 5)	DQ (5)	
pretomanid (Pa, 6)*	HZ (5)	
pyrazinamide (Z, 5)	MR (5)	
quabodepistat (Q, 5)	PaL (6)*	
rifampicin (R, 5)	RZ (5)	

* one animal from the treatment group developed repeated phenotypic resistance and was removed from subsequent analyses.

## Data Availability

PET/CT imaging data related to *M. tuberculosis*-infected marmosets have been deposited into Accessclinicaldata@NIAID with the link: https://accessclinicaldata.niaid.nih.gov/study-viewer/clinical_trials/MARM-TB-Drug-Regimens.
