# Supplementary material for: PET/CT imaging of tuberculosis lung lesions in marmosets treated with different drug regimens aligns with human clinical outcomes
Source: Sci Transl Med. Author manuscript; Available in PMC 2026 Feb 4. (PMC12870020; doi:10.1126/scitranslmed.ado9383)
Supplement: Supplemental Materials [file NIHMS2129178-supplement-Supplemental_Materials.pdf]

Supplementary materials

MATERIALS AND METHODS

Lesion histopathological classification

Individual lesions of the lung were photographed, measured, and preliminarily classified by their gross appearance. Formalin-fixed tissue sections were stained with hematoxylin and eosin and Masson's trichome for histologic confirmation. The lesions observed by imaging and collected were binned into three main types (*14, 34-36*). (1) Necrotic granulomas typically had grossly apparent caseation or liquefaction and central necrosis with an eosinophilic acellular region surrounded by epithelioid macrophages and further circumscribed with layers of fibrocytes histologically. (2) Cavitary lesions were defined as those lesions with either a radiologic or gross appearance of a central, empty (radiolucent) space bordered by sharply demarcated (radio-opaque) borders larger than a normal bronchiole in that region of the lung with or without liquefaction. Histologically, these spaces contained amorphic debris and appeared rimmed with foamy epithelioid macrophages, degenerate neutrophils, and further surrounded by fibrous connective tissue often replacing regions of bronchial wall. Often these lesions are surrounded by granulomatous inflammation. A third category was (3) cellular/fibrotic or non-necrotizing granulomas. At necropsy, these lesions presented as solid masses lacking obvious caseation or liquefaction, and on histologic examination, they contained numerous neutrophils and epithelioid macrophages and occasional multinucleated giant cells but lacked necrotic material. This core was often surrounded by lymphocytes and fibrous connective tissue, sometimes with invasive non-necrotic granulomatous inflammation. Some showed fewer macrophages and neutrophils and a predominance of fibrocytes.

PET/CT data analysis

For each animal, the 6 PET/CT files were co-registered and aligned in MIM Maestro (v. 7, MIM Software Inc., Cleveland, Ohio), and the lesions were captured into a three-dimensional region of interest (ROI) by 3 readers as previously described (*33*). The lesion ROIs were individually adjusted to encapsulate the lesion volume on each serial scan and the numerical output for the lesion features, including volume, mean HU, radiodense volume (-100 to 200 HU), and soft volume (-500 to -100 HU) from the CT images were exported. For the PET scans, the CT ROIs were transferred to the aligned PET scans, and features like max SUV, mean SUV, and total FDG uptake for each ROI were extracted. The data used for assessment of treatment response are an average of readings for each feature. The CFU and histopathology data for each lesion were mapped to the extracted PET/CT data for each lesion to estimate the lesion responses during the initial 4 weeks and continuation or final 4 weeks of treatment.

Modeling of bedaquiline dose

Pharmacokinetic data from 13 marmosets was available for modeling. The marmosets received doses of either 7 or 20 mg/kg. Marmosets were dosed either daily, or three times per week, or daily for 15-16 days followed by three times a week. Samples for PK analysis were collected at different times depending on the dosing frequency including 3, 4, 6, 7, 24, 48, and 72 hours post-dose. In total, 240 samples were collected and analyzed, comprising 120 samples for bedaquiline PK and 120 for its metabolite.

The population PK of bedaquiline and its metabolite was described using nonlinear mixed-effect modeling with the software NONMEM version 7.4.4 (Icon Development Solutions, Ellicott City, MD) and the first-order conditional estimation with interaction (FOCE-I) algorithm. R software version 3.2.5 was used for the graphical processing of NONMEM output and managing

the model development process. The modeling process was conducted stepwise. Firstly, a structural model was developed to describe plasma concentrations of bedaquiline parent. One-, two-, and three-compartment models with first-order elimination were evaluated, along with first-order absorption with and without a time lag. Allometric scaling was employed to adjust for the effect of body size on disposition parameters, with allometric exponents fixed to 0.75 for clearance parameters and 1 for volumes of distribution, as advocated by Anderson and Holford (*37*). Log-normally distributed random effects were included for the disposition parameters to account for between-subject variability (BSV), and for absorption parameters to describe between-occasion variability (BOV). The typical value of oral bioavailability parameter was fixed to 1. Secondly, the model was expanded to include the disposition of the metabolite in plasma, and one- and two-compartment models with first-order elimination were evaluated. The performance of the final model was assessed using goodness-of-fit plots and a visual predictive check (VPC).

The final model was used to perform simulations using the R package mrgSolve to predict dosing regimens for marmosets that are expected to achieve exposures equivalent to humans receiving the standard bedaquiline treatment of two weeks 400 mg of daily doses, followed by 200 mg three times per week. We used the exposure values reported by Svensson et al. for bedaquiline administered without ART (*38*). Since the metabolite M2 is active against M. tuberculosis but approximately 5-fold less potent than bedaquiline, we calculated the equivalent effective concentration using the formula below

$$C_{equivalent} = C_{bedaquiline} + \frac{C_{M2}}{5}$$

where $C_{equivalent}$ denotes the equivalent effective concentration, $C_{bedaquiline}$ represents the bedaquiline concentration, and C_{M2} the concentration of M2.

The pharmacokinetics of bedaquiline in plasma was well described using a three-compartment disposition model with first-order absorption and elimination, and its M2 was described by another one-compartment model, as shown in **Figure S18**. The final parameter estimates are provided in **Table S5**. For a typical 0.41-kg marmoset, the estimated clearances of bedaquiline and its metabolite M2 were 0.046 L/h and 0.0362 L/h, respectively. A visual predictive check of the model is available in **Figure S19**, showing a satisfactory fit of the model to the data.

We observed that, at steady state, the parent-to-metabolite concentration ratio in marmosets is about 1 to 2, hence the contribution of M2 to the killing of the bacteria is not negligible. This is contrary to what is observed in humans, where the steady-state parent-to-metabolite concentration ratio is flipped the other way, ~4 to 1 ratio (*38*).

Simulations using this model suggested a loading dose of 8 mg/kg for 2 weeks followed by a maintenance dose of 6 mg/kg achieved equivalent concentrations to those observed in humans (*38*), as shown in **Figure S20**.

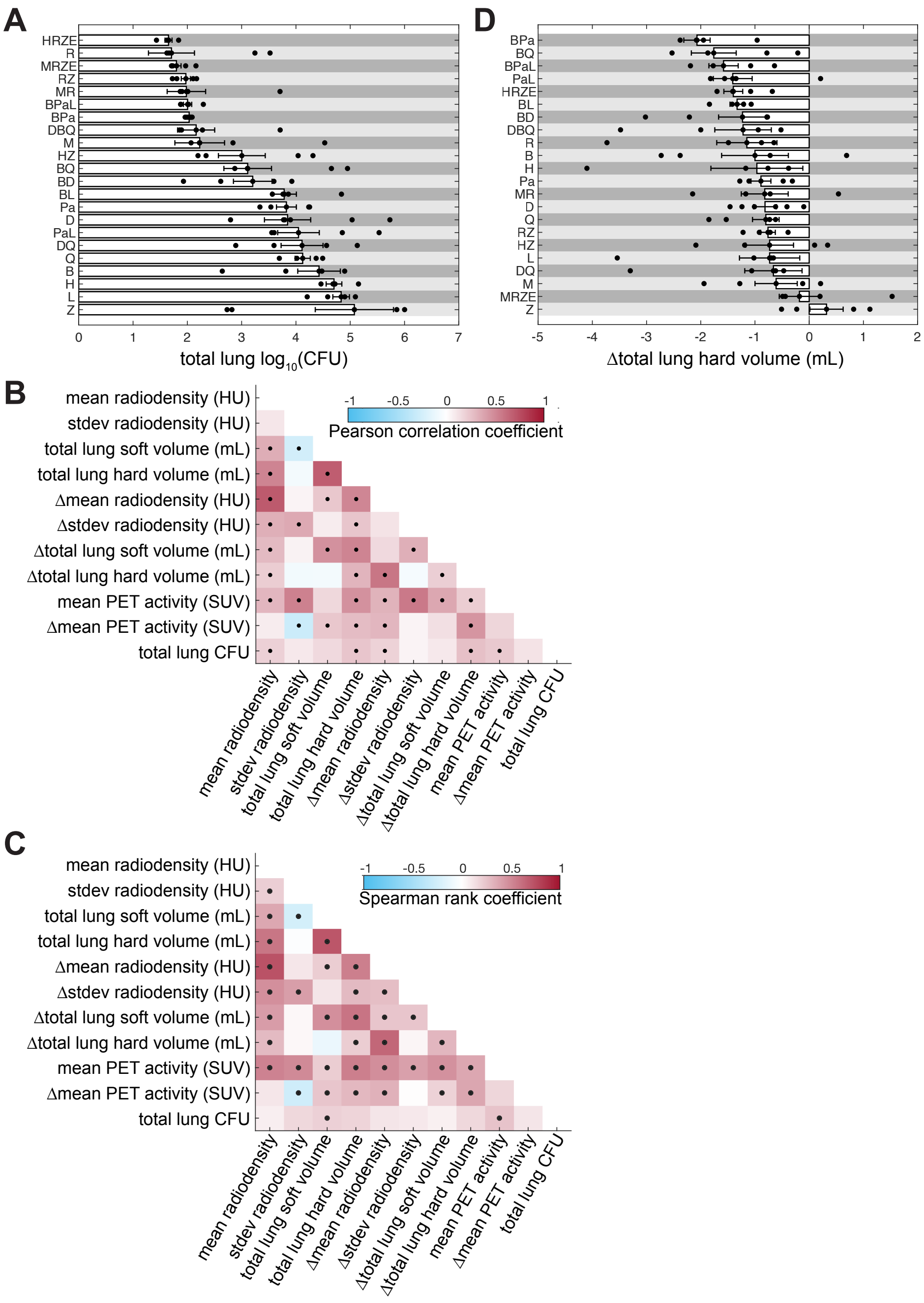

Figure S1. **PET/CT biomarkers and bacterial burden do not correlate**. (A) log10 transformed total lung CFU. Each dot represents an animal within treatment group. Bars represent the median across animals within treatment group. Error bars represent the standard error of the mean. (B) Pearson correlation coefficients for each possible pairing of PET/CT biomarkers and total lung CFU across all animals. Dots indicate statistical significance ($p<0.05$) of the correlation strength. All correlation coefficients are > -0.75 and < 0.75. (C) Spearman rank correlation coefficients for each possible pairing

of PET/CT biomarkers and total lung CFU across all animals. Dots indicate statistical significance ($p<0.05$) of the correlation strength. All correlation coefficients are > -0.75 and < 0.75. (D) Change in total lung hard volume from the start of treatment to the end of treatment (2 months) per animal. Bars represent the median across animals within treatment group. Error bars represent the standard error of the mean.

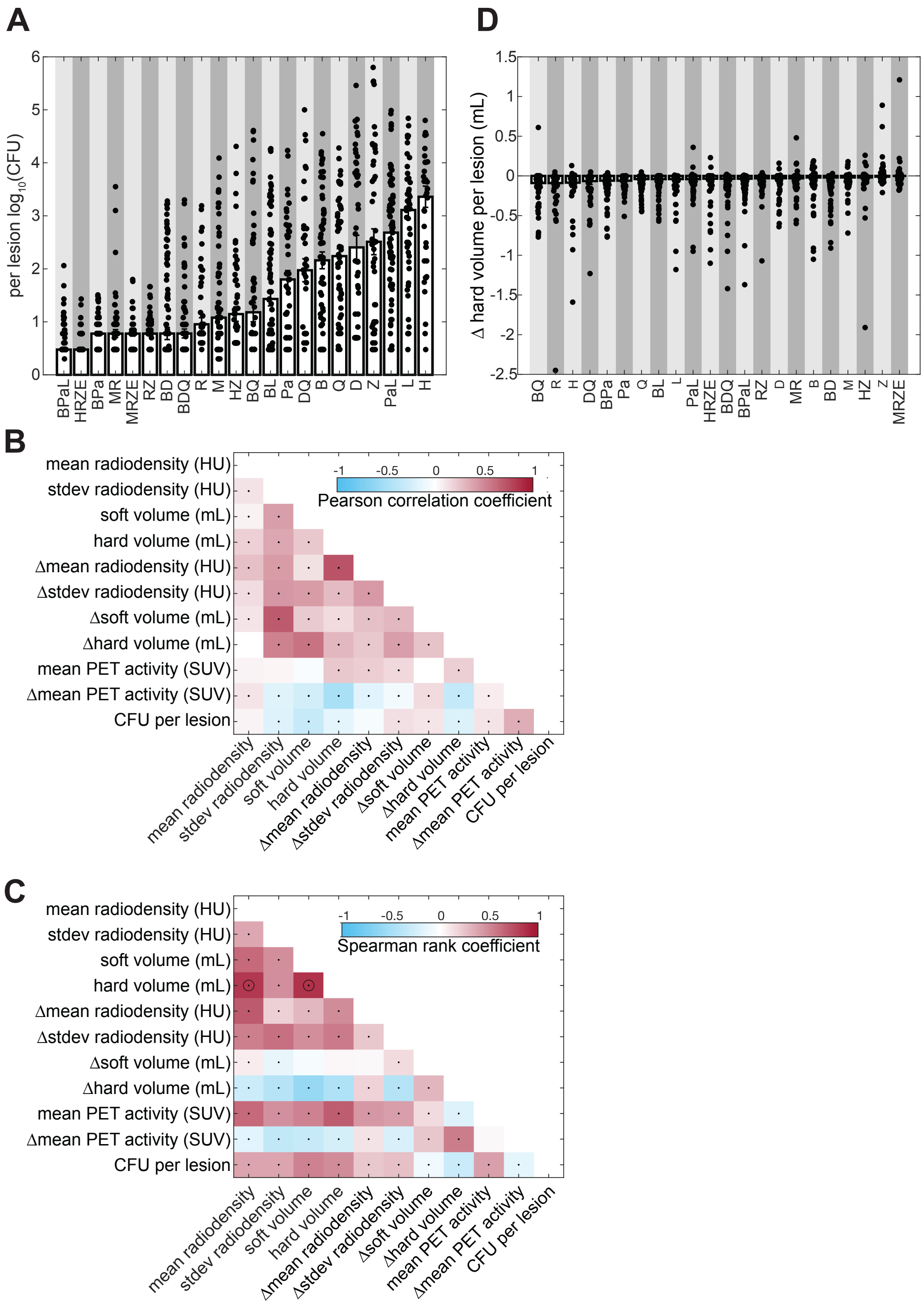

Figure S2. **PET/CT biomarkers and bacterial burden do not correlate.** (A) log10 transformed CFU per lesion. Each dot represents a lesion within treatment group. Bars represent the median across all lesions from all the animals from that treatment group. Error bars represent the standard error of the mean. (B) Pearson correlation coefficients for each possible pairing of PET/CT biomarkers and per-lesion CFU across all lesions. Dots indicate statistical significance ($p<0.05$) of the correlation strength. All

correlation coefficients are > -0.75 and < 0.75. (C) Spearman rank correlation coefficients for each possible pairing of PET/CT biomarkers and total lung CFU across all animals. Dots indicate statistical significance ($p<0.05$) of the correlation strength. Circles indicate correlation coefficients > 0.75 or < -0.75. (D) Change in per-lesion hard volume from the start of treatment to the end of treatment (2 months). Bars represent the median across all lesions within treatment group. Error bars represent the standard error of the mean.

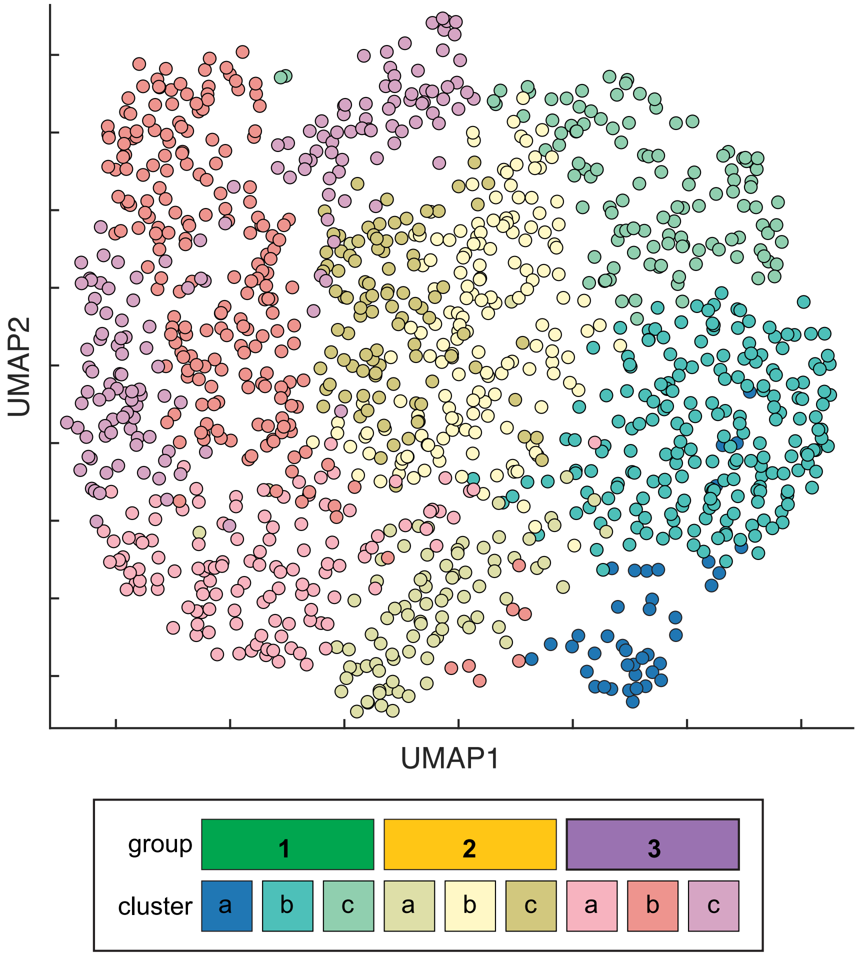

Figure S3. Distribution of clusters across all lesions. Leiden community detection was used to perform unsupervised clustering of lesions using PET/CT values after eight weeks of treatment, change in PET/CT values from the start of treatment (0 weeks) to the end of treatment (8 weeks, complete treatment), and bacterial burden per lesion at the cessation of treatment (8 weeks) as input features. Cluster colors correspond to those defined in Figure 1.

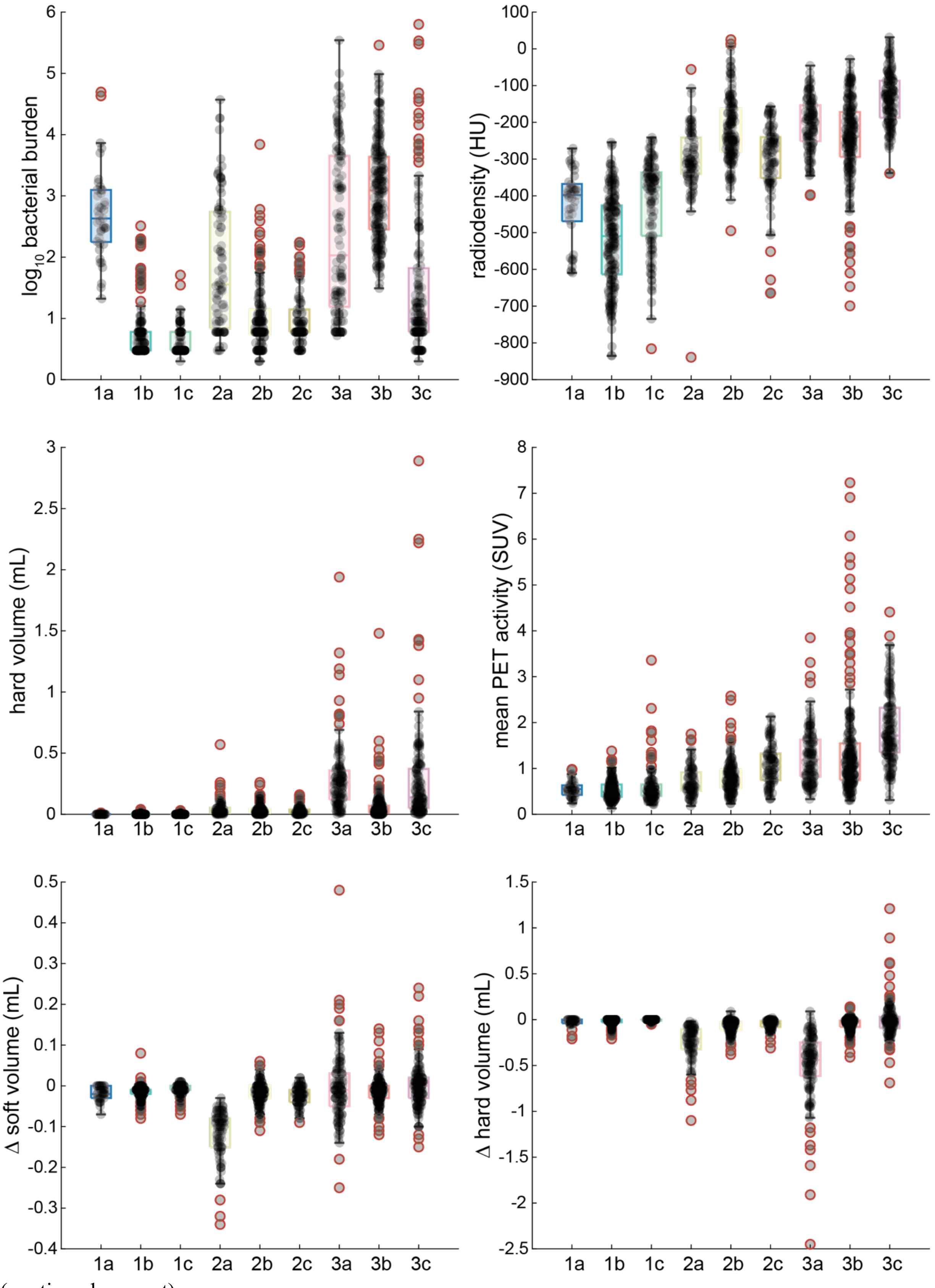

(continued on next)

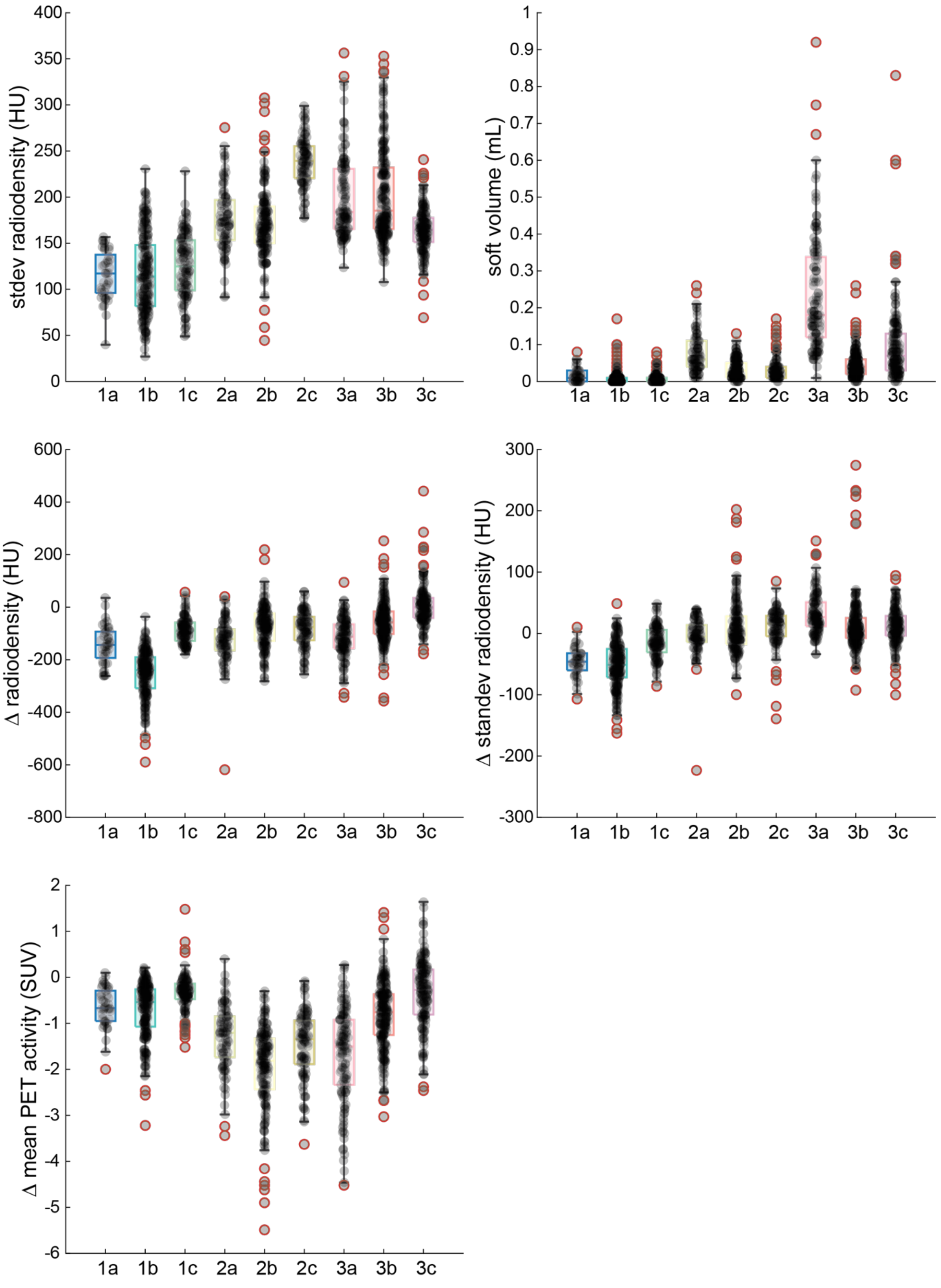
stdev radiodensity (HU)
soft volume (mL)
Δ radiodensity (HU)
Δ standev radiodensity (HU)
Δ mean PET activity (SUV)
1a 1b 1c 2a 2b 2c 3a 3b 3c

Figure S4. Per-lesion features that define the complete treatment clusters. Box plots depict the per-lesion features that define each cluster. Red outlines are outliers, values more than 1.5x IQR (interquartile range). Cluster labels correspond to those defined in Figure 1.

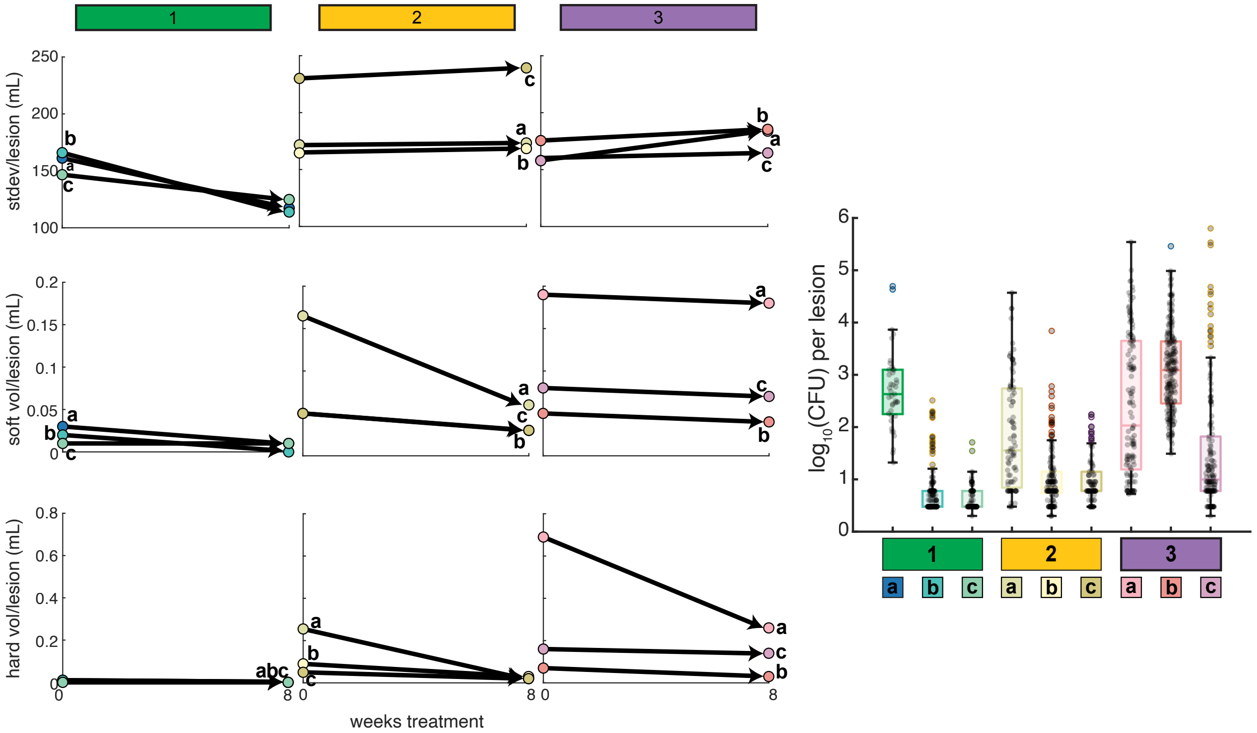

Figure S5. Additional features that define the complete treatment clusters. (A) Median feature values that characterize each lesion cluster at the start and end (8 weeks) of treatment and (B) per-lesion bacterial burden distribution at necropsy across clusters. Colors correspond to those defined in Figure 1.

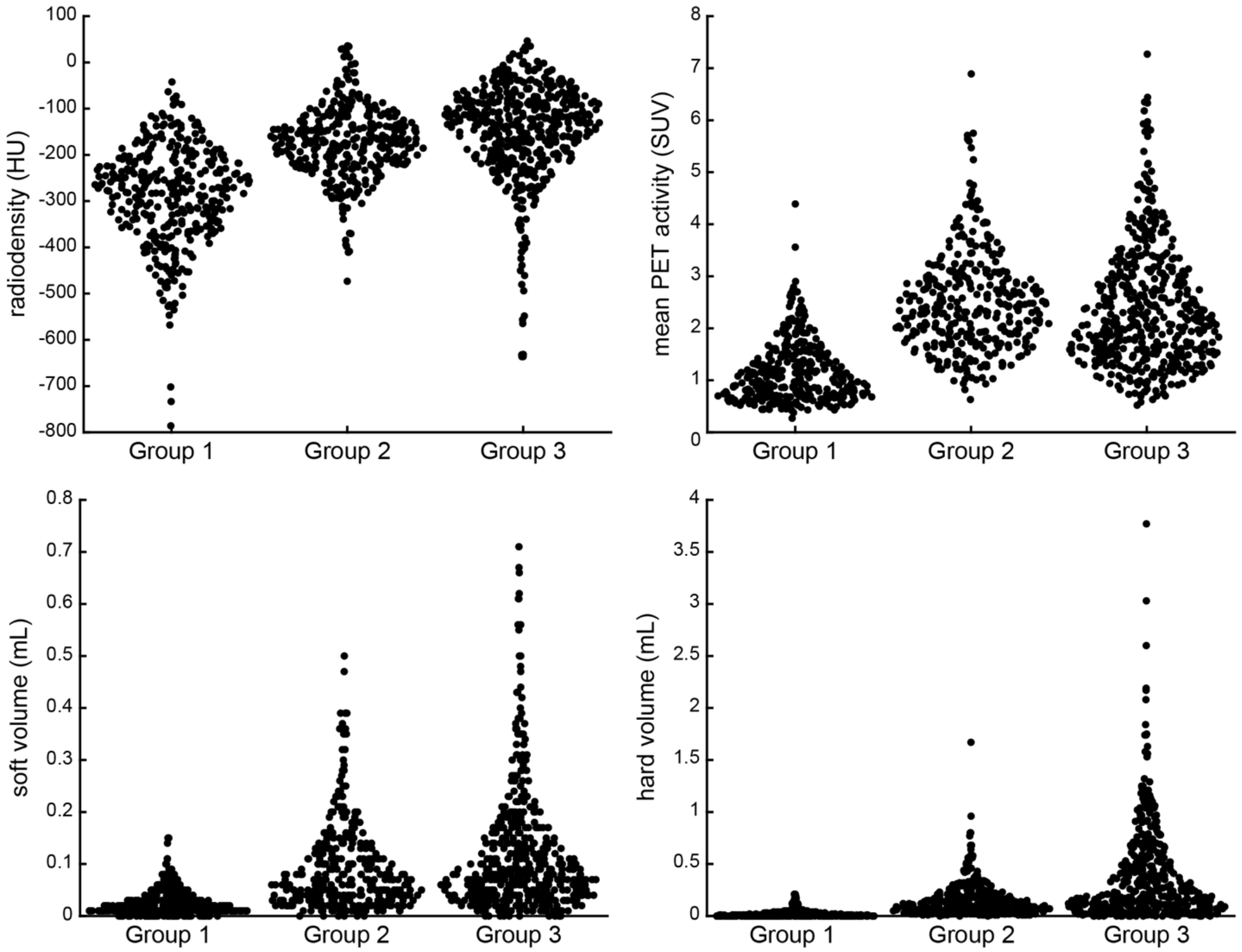

Figure S6. Distributions of per-lesion features at start of treatment across clustered groups. PET and CT features at start of treatment from lesions that clustered in groups 1, 2, and 3, respectively, using terminal features and changes in features from start to end of treatment as clustering variables.

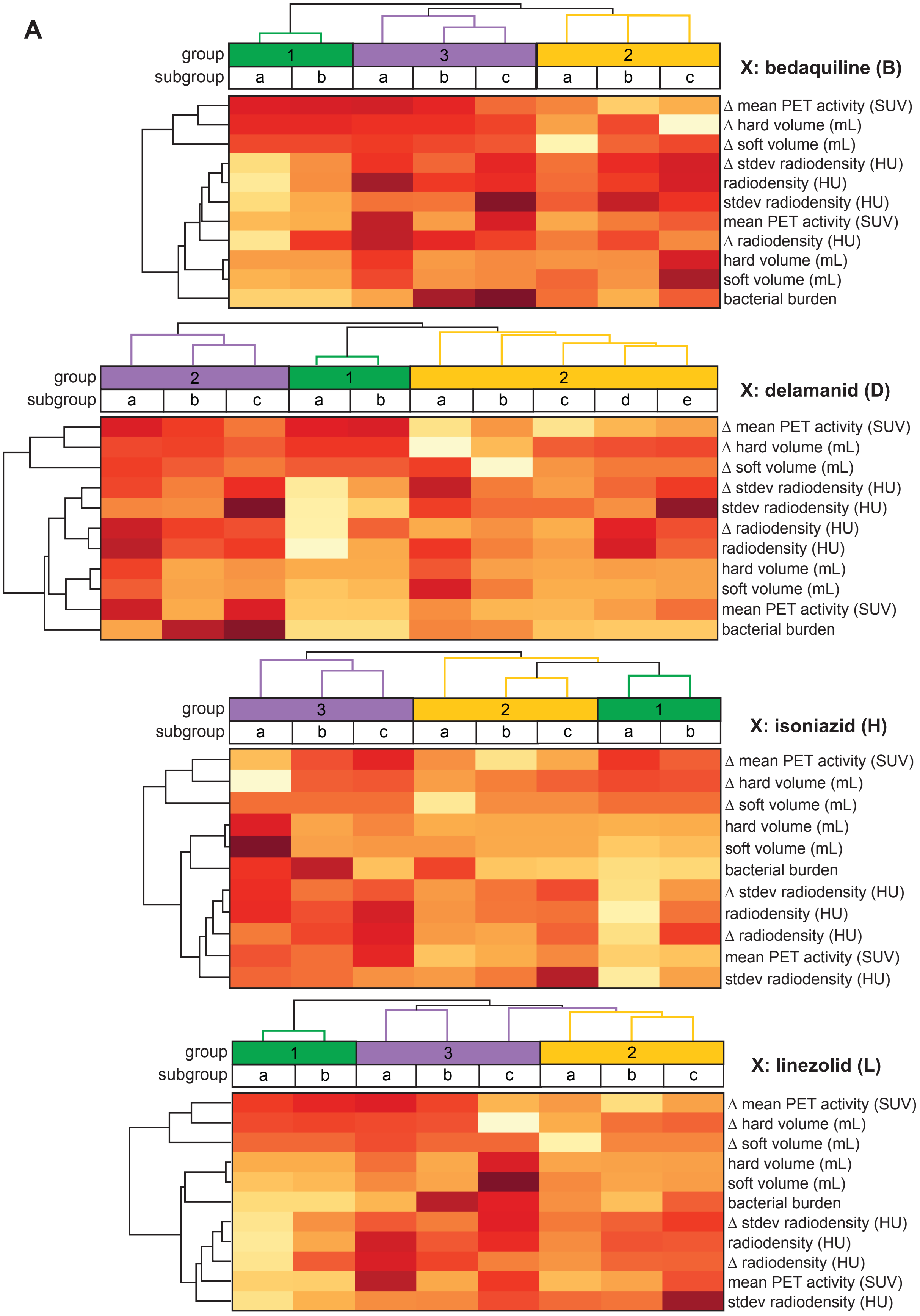
A
group
subgroup
1
3
2
a
b
a
b
c
a
b
c
X: bedaquiline (B)
Δ mean PET activity (SUV)
Δ hard volume (mL)
Δ soft volume (mL)
Δ stdev radiodensity (HU)
radiodensity (HU)
stdev radiodensity (HU)
mean PET activity (SUV)
Δ radiodensity (HU)
hard volume (mL)
soft volume (mL)
bacterial burden
group
subgroup
2
1
2
a
b
c
a
b
a
b
c
d
e
X: delamanid (D)
Δ mean PET activity (SUV)
Δ hard volume (mL)
Δ soft volume (mL)
Δ stdev radiodensity (HU)
stdev radiodensity (HU)
Δ radiodensity (HU)
radiodensity (HU)
hard volume (mL)
soft volume (mL)
mean PET activity (SUV)
bacterial burden
group
subgroup
3
2
1
a
b
c
a
b
c
a
b
X: isoniazid (H)
Δ mean PET activity (SUV)
Δ hard volume (mL)
Δ soft volume (mL)
hard volume (mL)
soft volume (mL)
bacterial burden
Δ stdev radiodensity (HU)
radiodensity (HU)
Δ radiodensity (HU)
mean PET activity (SUV)
stdev radiodensity (HU)
group
subgroup
1
3
2
a
b
a
b
c
a
b
c
X: linezolid (L)
Δ mean PET activity (SUV)
Δ hard volume (mL)
Δ soft volume (mL)
hard volume (mL)
soft volume (mL)
bacterial burden
Δ stdev radiodensity (HU)
radiodensity (HU)
Δ radiodensity (HU)
mean PET activity (SUV)
stdev radiodensity (HU)

A (continued)

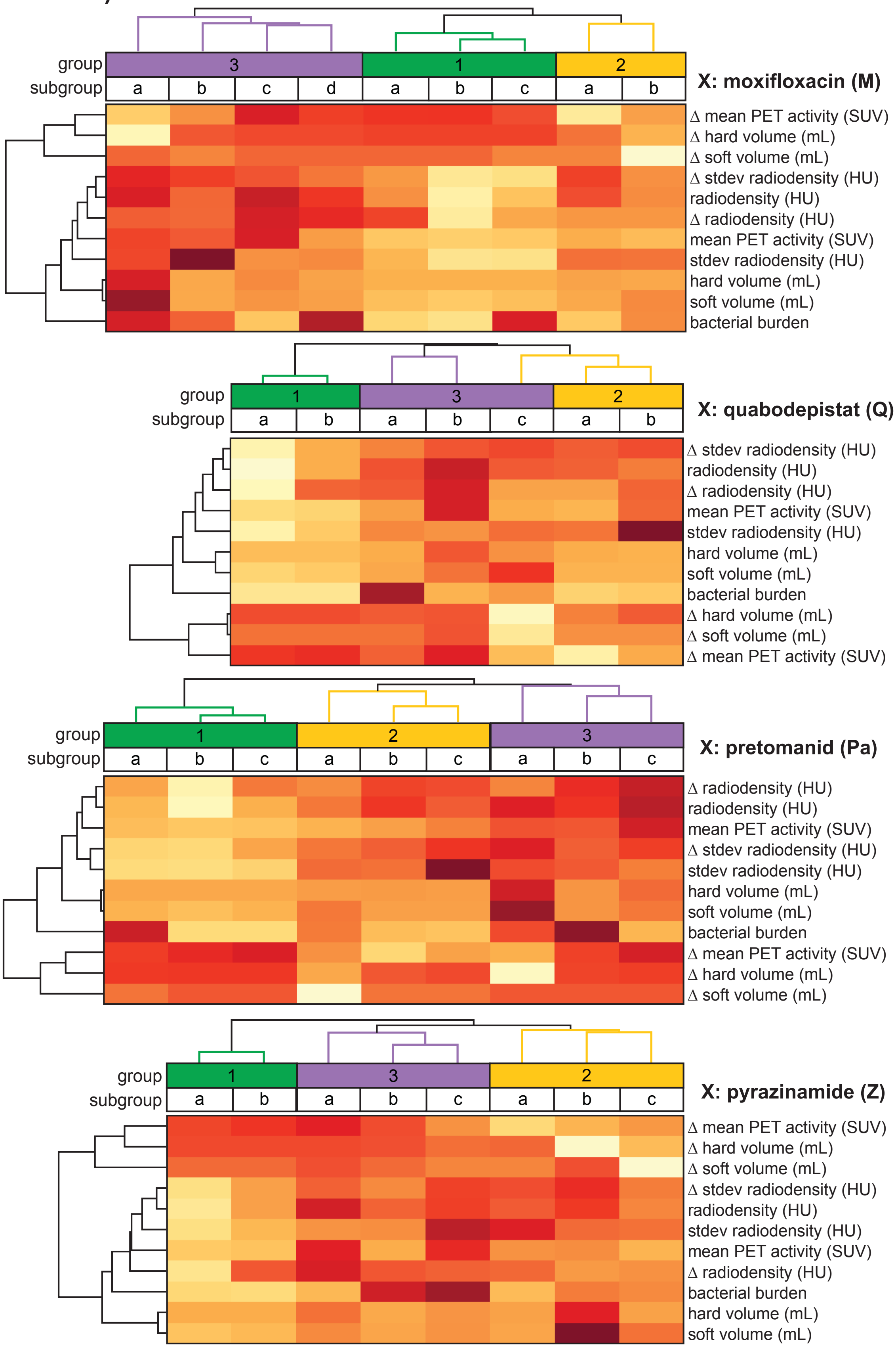
group
subgroup
3 1 2
a b c d a b c a b
X: moxifloxacin (M)
Δ mean PET activity (SUV)
Δ hard volume (mL)
Δ soft volume (mL)
Δ stdev radiodensity (HU)
radiodensity (HU)
Δ radiodensity (HU)
mean PET activity (SUV)
stdev radiodensity (HU)
hard volume (mL)
soft volume (mL)
bacterial burden
group
subgroup
1 3 2
a b a b c a b
X: quabodepistat (Q)
Δ stdev radiodensity (HU)
radiodensity (HU)
Δ radiodensity (HU)
mean PET activity (SUV)
stdev radiodensity (HU)
hard volume (mL)
soft volume (mL)
bacterial burden
Δ hard volume (mL)
Δ soft volume (mL)
Δ mean PET activity (SUV)
group
subgroup
1 2 3
a b c a b c a b c
X: pretomanid (Pa)
Δ radiodensity (HU)
radiodensity (HU)
mean PET activity (SUV)
Δ stdev radiodensity (HU)
stdev radiodensity (HU)
hard volume (mL)
soft volume (mL)
bacterial burden
Δ mean PET activity (SUV)
Δ hard volume (mL)
Δ soft volume (mL)
group
subgroup
1 3 2
a b a b c a b c
X: pyrazinamide (Z)
Δ mean PET activity (SUV)
Δ hard volume (mL)
Δ soft volume (mL)
Δ stdev radiodensity (HU)
radiodensity (HU)
stdev radiodensity (HU)
mean PET activity (SUV)
Δ radiodensity (HU)
bacterial burden
hard volume (mL)
soft volume (mL)

A (continued)

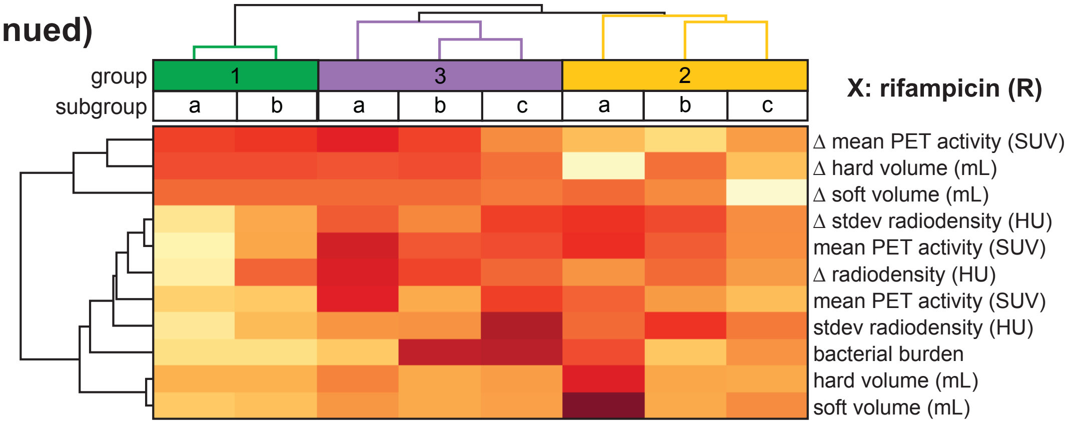

B

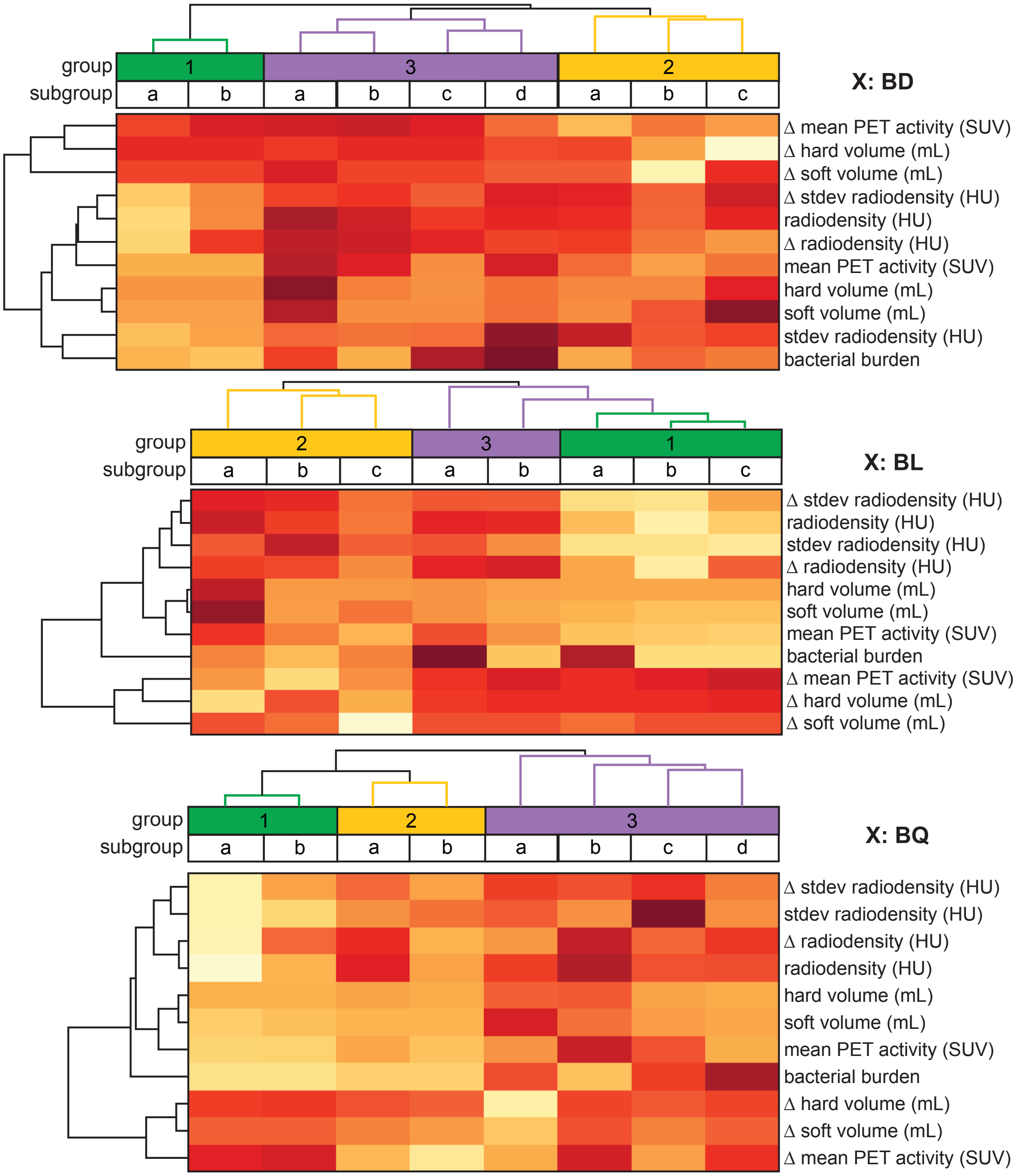

B (continued)

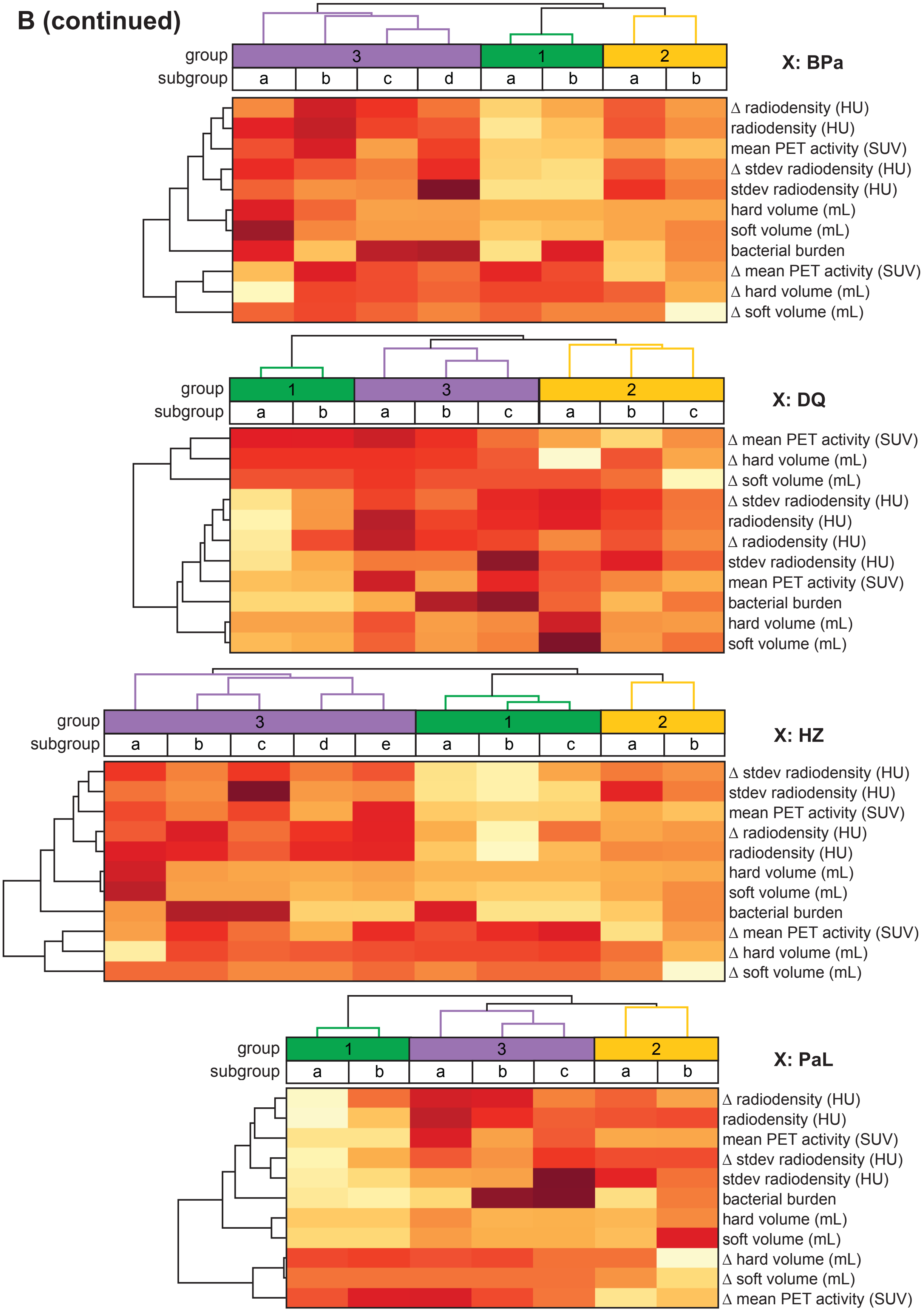
X: BPa
group
subgroup
3
1
2
a
b
c
d
a
b
a
b
Δ radiodensity (HU)
radiodensity (HU)
mean PET activity (SUV)
Δ stdev radiodensity (HU)
stdev radiodensity (HU)
hard volume (mL)
soft volume (mL)
bacterial burden
Δ mean PET activity (SUV)
Δ hard volume (mL)
Δ soft volume (mL)
X: DQ
group
subgroup
1
3
2
a
b
a
b
c
a
b
c
Δ mean PET activity (SUV)
Δ hard volume (mL)
Δ soft volume (mL)
Δ stdev radiodensity (HU)
radiodensity (HU)
Δ radiodensity (HU)
stdev radiodensity (HU)
mean PET activity (SUV)
bacterial burden
hard volume (mL)
soft volume (mL)
X: HZ
group
subgroup
3
1
2
a
b
c
d
e
a
b
c
a
b
Δ stdev radiodensity (HU)
stdev radiodensity (HU)
mean PET activity (SUV)
Δ radiodensity (HU)
radiodensity (HU)
hard volume (mL)
soft volume (mL)
bacterial burden
Δ mean PET activity (SUV)
Δ hard volume (mL)
Δ soft volume (mL)
X: PaL
group
subgroup
1
3
2
a
b
a
b
c
a
b
Δ radiodensity (HU)
radiodensity (HU)
mean PET activity (SUV)
Δ stdev radiodensity (HU)
stdev radiodensity (HU)
bacterial burden
hard volume (mL)
soft volume (mL)
Δ hard volume (mL)
Δ soft volume (mL)
Δ mean PET activity (SUV)

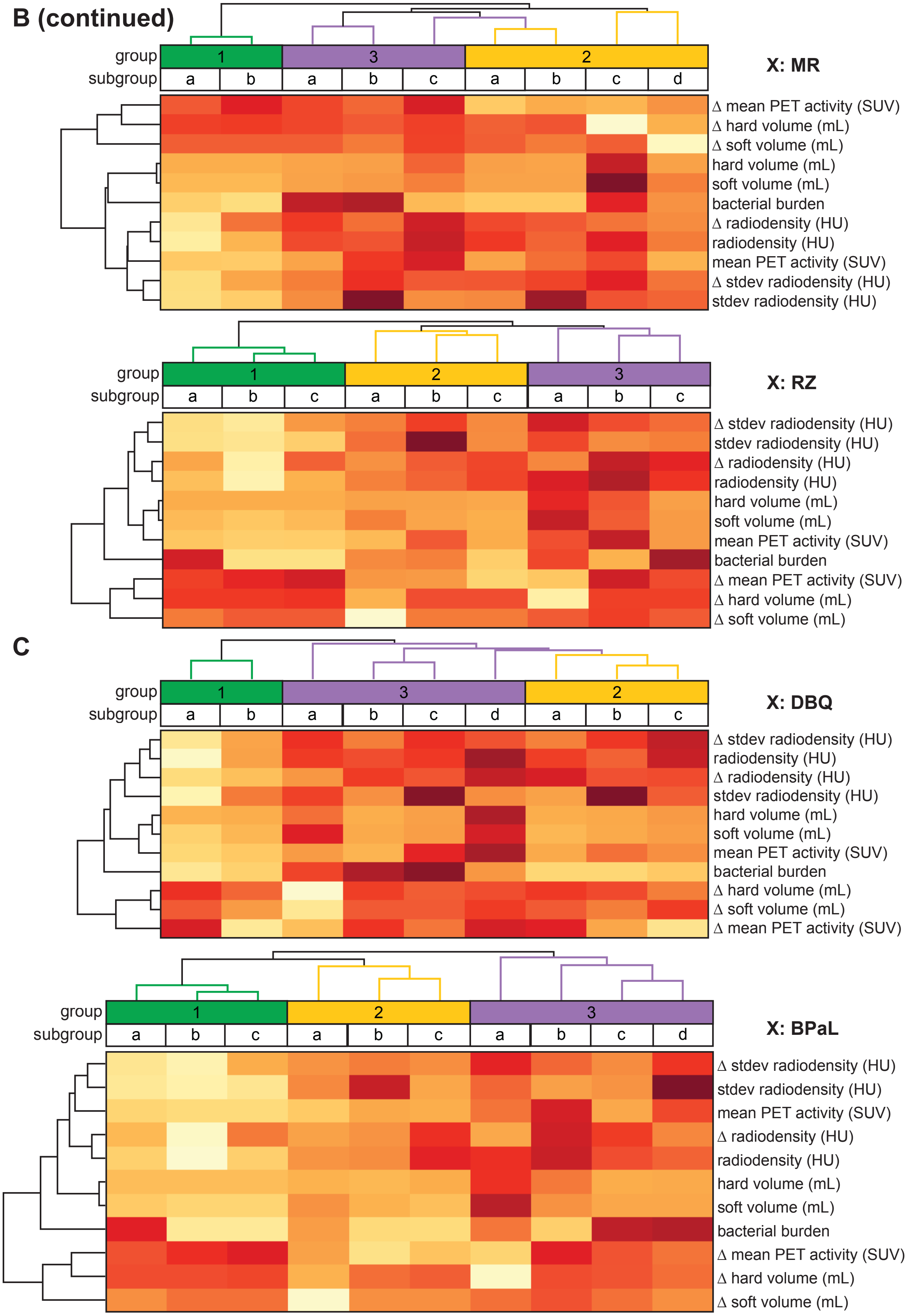
B (continued)
group
subgroup
1 3 2
a b a b c a b c d
X: MR
Δ mean PET activity (SUV)
Δ hard volume (mL)
Δ soft volume (mL)
hard volume (mL)
soft volume (mL)
bacterial burden
Δ radiodensity (HU)
radiodensity (HU)
mean PET activity (SUV)
Δ stdev radiodensity (HU)
stdev radiodensity (HU)
group
subgroup
1 2 3
a b c a b c a b c
X: RZ
Δ stdev radiodensity (HU)
stdev radiodensity (HU)
Δ radiodensity (HU)
radiodensity (HU)
hard volume (mL)
soft volume (mL)
mean PET activity (SUV)
bacterial burden
Δ mean PET activity (SUV)
Δ hard volume (mL)
Δ soft volume (mL)
C
group
subgroup
1 3 2
a b a b c d a b c
X: DBQ
Δ stdev radiodensity (HU)
radiodensity (HU)
Δ radiodensity (HU)
stdev radiodensity (HU)
hard volume (mL)
soft volume (mL)
mean PET activity (SUV)
bacterial burden
Δ hard volume (mL)
Δ soft volume (mL)
Δ mean PET activity (SUV)
group
subgroup
1 2 3
a b c a b c a b c d
X: BPaL
Δ stdev radiodensity (HU)
stdev radiodensity (HU)
mean PET activity (SUV)
Δ radiodensity (HU)
radiodensity (HU)
hard volume (mL)
soft volume (mL)
bacterial burden
Δ mean PET activity (SUV)
Δ hard volume (mL)
Δ soft volume (mL)

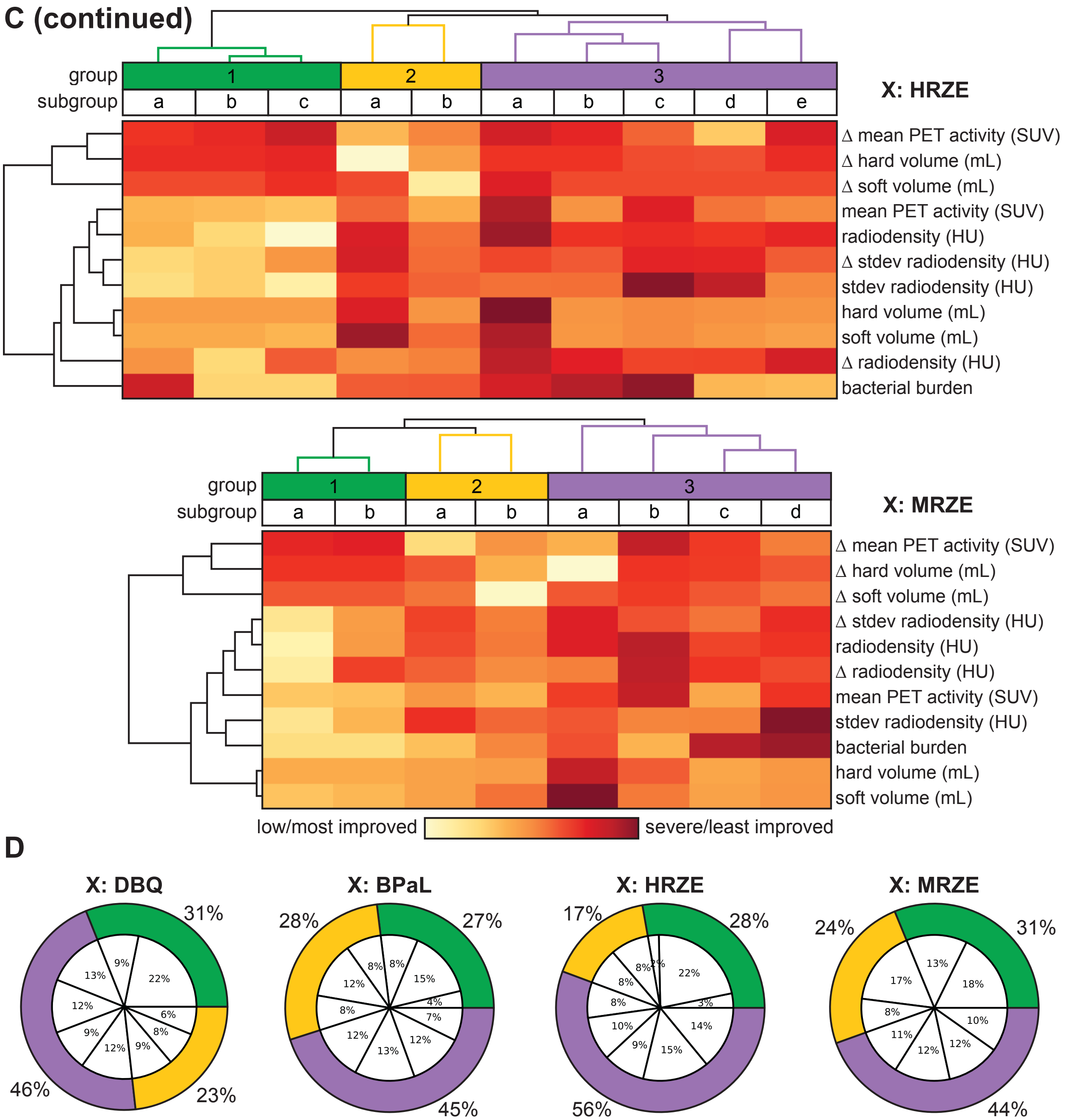

Figure S7. Feature profiles for lesion clusters from leave-one-out analyses. Leiden community detection (LCD) was performed 22 times, each time leaving out one of the 22 treatment arms. Median feature values across the resultant clusters were used in hierarchical clustering and displayed in heatmaps to identify patterns across the clusters similar to those identified in Figure 1. Three groups (1, 2, 3) of similar clusters are labeled from the hierarchical clustering, and clusters are labeled (a, b, c) within each group. Feature values were scaled for visualization. The severe/low color scale is relative to the values in

this visualization (not absolute). Δ refers to changes in feature from the start (0 weeks) to EOT (8 weeks). X: indicates which treatment arm was left out for each heatmap and pie chart. Resolution for LCD was 0.6 unless otherwise indicated; resolution was varied to achieve the most interpretable clusters. (A) Analyses leaving out each of the monotherapy treatment arms. Isoniazid and pretomanid clustering resolutions were 0.55; quabodepistat resolution was 0.5. (B) Analyses leaving out pairwise treatment arms. BQ, BPa, DQ, and PaL clustering resolutions were 0.55. (C) Analyses leaving out higher-order treatment arms. BPaL clustering resolution was 0.7. (D) Distributions of the groupings from clustering from the leave-one-out analyses that left out higher-order combinations. The distribution of all 22 treatments was 41%/29%/30% (purple/yellow/green) (Figure 2A). The pie charts in (D) show that the distributions across those three groups are similar when each of the higher-order combinations is removed from the set of treatment arms. There is a slight shift, as expected, to a greater proportion of purple and lower yellow/green; the higher-order combinations typically resulted in more improvement, so their removal resulted in a higher proportion of severe lesions across the total population.

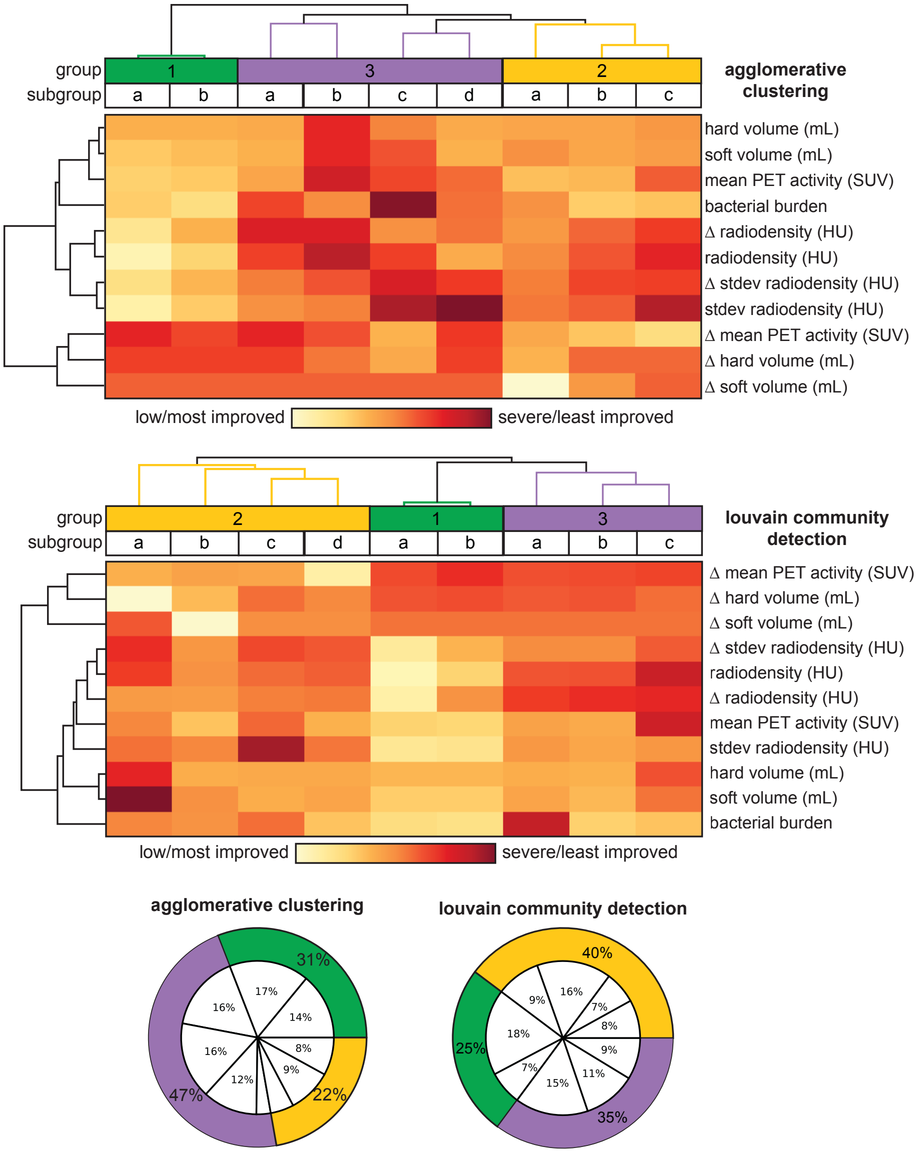

Figure S8. Feature profiles for lesion clusters from additional clustering algorithms. Clustering was performed using agglomerative clustering (*sklearn*) and Louvain community detection (*scanpy*, default parameters). Median feature values across the resultant clusters were used in hierarchical clustering and displayed in heatmaps to identify patterns across the clusters similar to those identified in Figure 1. Three groups (1, 2, 3) of similar clusters are labeled from the hierarchical clustering, and clusters are labeled (a, b, c) within each group. Feature values were scaled for visualization. The severe/low color scale is relative to the values in this visualization (not absolute). Δ refers to changes in feature from the start (0 weeks) to EOT (8 weeks). Pie charts display the distributions of the clusters and groupings from the two algorithms.

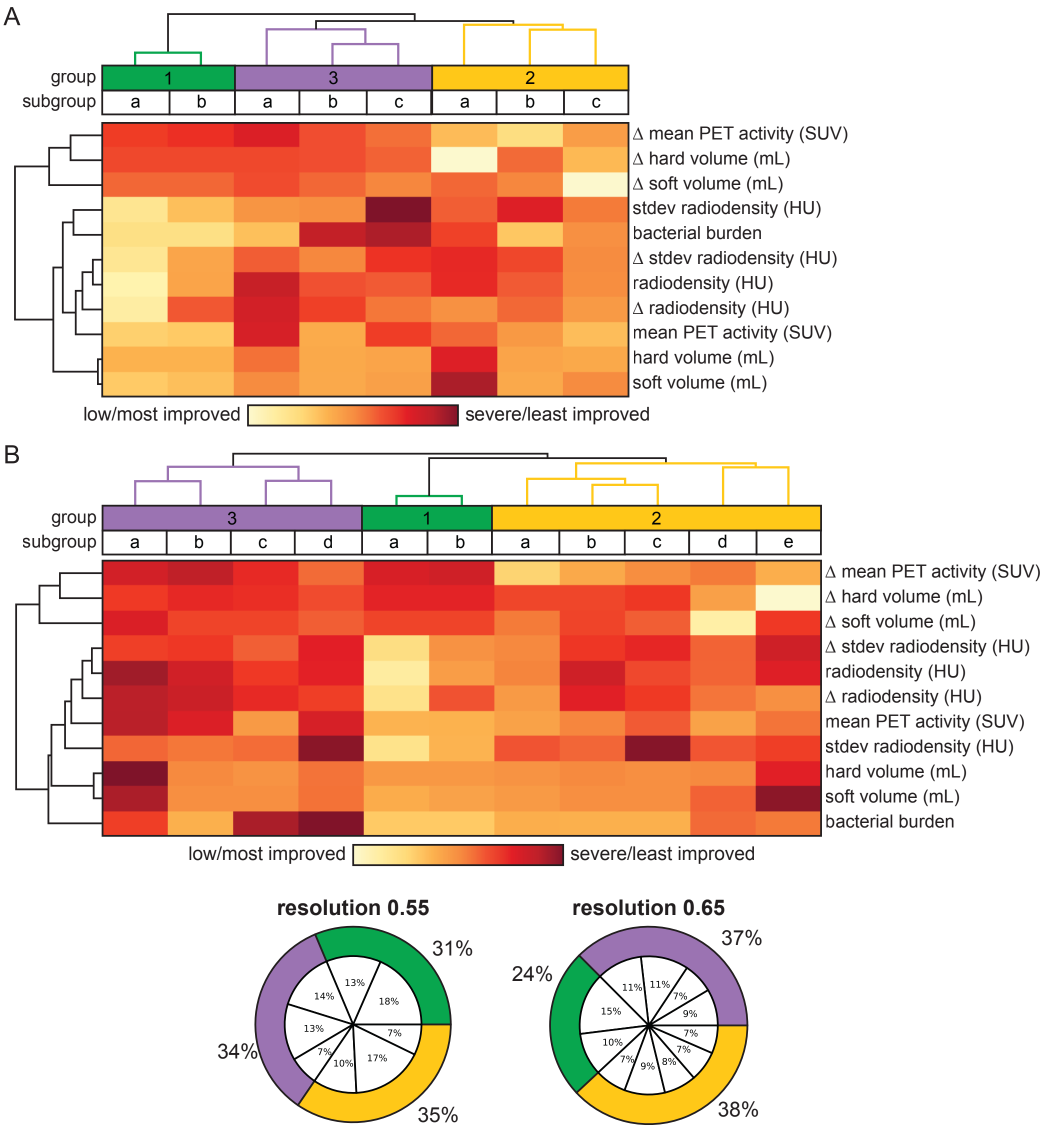

Figure S9. Feature profiles for lesion clusters from varied resolution of Leiden community detection. Clustering was performed using Leiden community detection at resolution 0.55 (A) and 0.65 (B). Median feature values across the resultant clusters were used in hierarchical clustering and displayed in heatmaps to identify patterns across the clusters similar to those identified in Figure 1. Three groups (1, 2, 3) of similar clusters are labeled from the hierarchical clustering, and clusters are labeled (a, b, c) within each group. Feature values were scaled for visualization. The severe/low color scale is relative to the values in this visualization (not absolute). Δ refers to changes in feature from the start (0 weeks) to EOT (8 weeks). Pie charts display the distributions of the clusters and groupings from the resolutions.

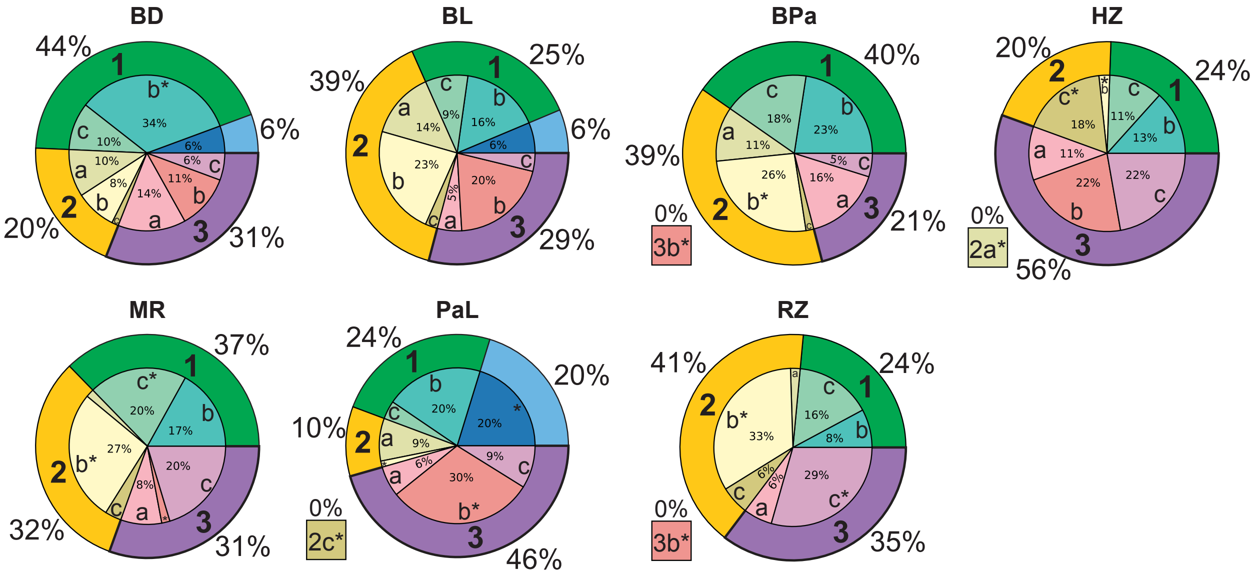

Figure S10. Distribution of complete treatment clusters across lesions. Inner pie charts represent the distribution of the clusters from complete treatment (two months) analysis across individual drug treatments. Cluster groups are indicated in the outer pie charts. 1: low CFU and low/improved PET/CT pathology; 2: low or varied CFU and moderate/improved PET/CT pathology; 3: severe/worsened PET/CT pathology and high CFU, except 3c. Wedge colors correspond to those in Figure 2. * indicates significant deviation (95% confidence interval) from the expected (average) number of lesions in a specific cluster by adjusted residual analysis following the chi-squared test for homogeneity ($p < 0.001$). Boxes to the left of each pie chart indicate significant underrepresentation (complete absence) from specified clusters. Unlabeled inner slices are <5% of the total for that drug treatment.

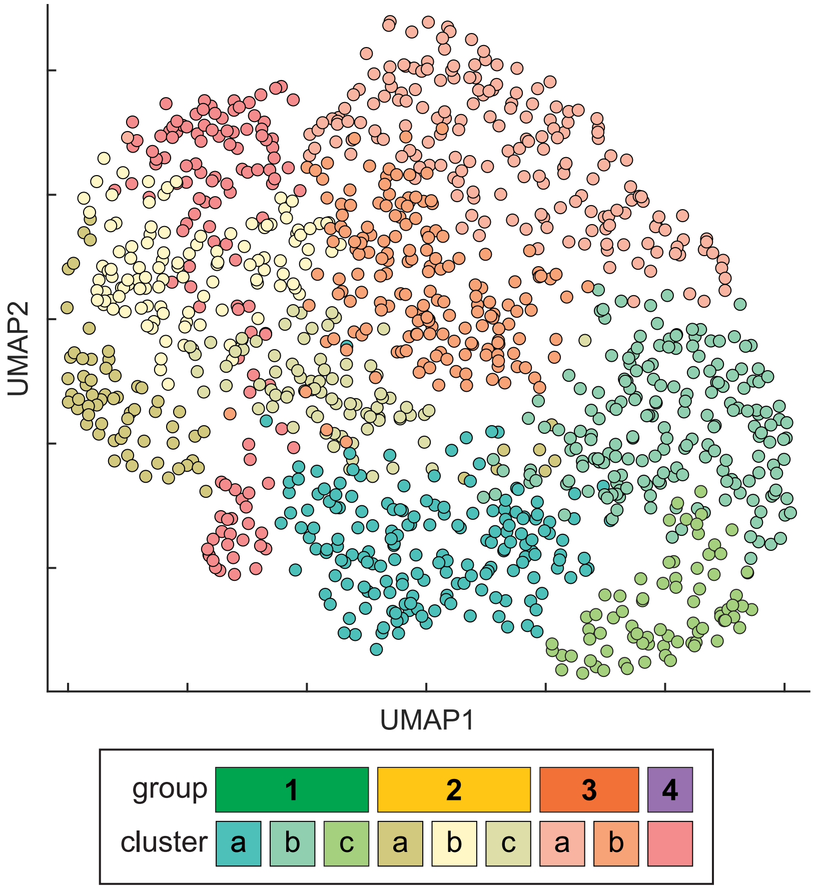

Figure S11. Distribution of continuation phase clusters across all lesions. Leiden community detection was used to perform unsupervised clustering of lesions using PET/CT values after eight weeks of treatment, change in PET/CT values from the middle of treatment (4 weeks) to the end of treatment (8 weeks), and bacterial burden per lesion at the cessation of treatment (8 weeks) as input features. Cluster colors correspond to those defined in Figure 3.

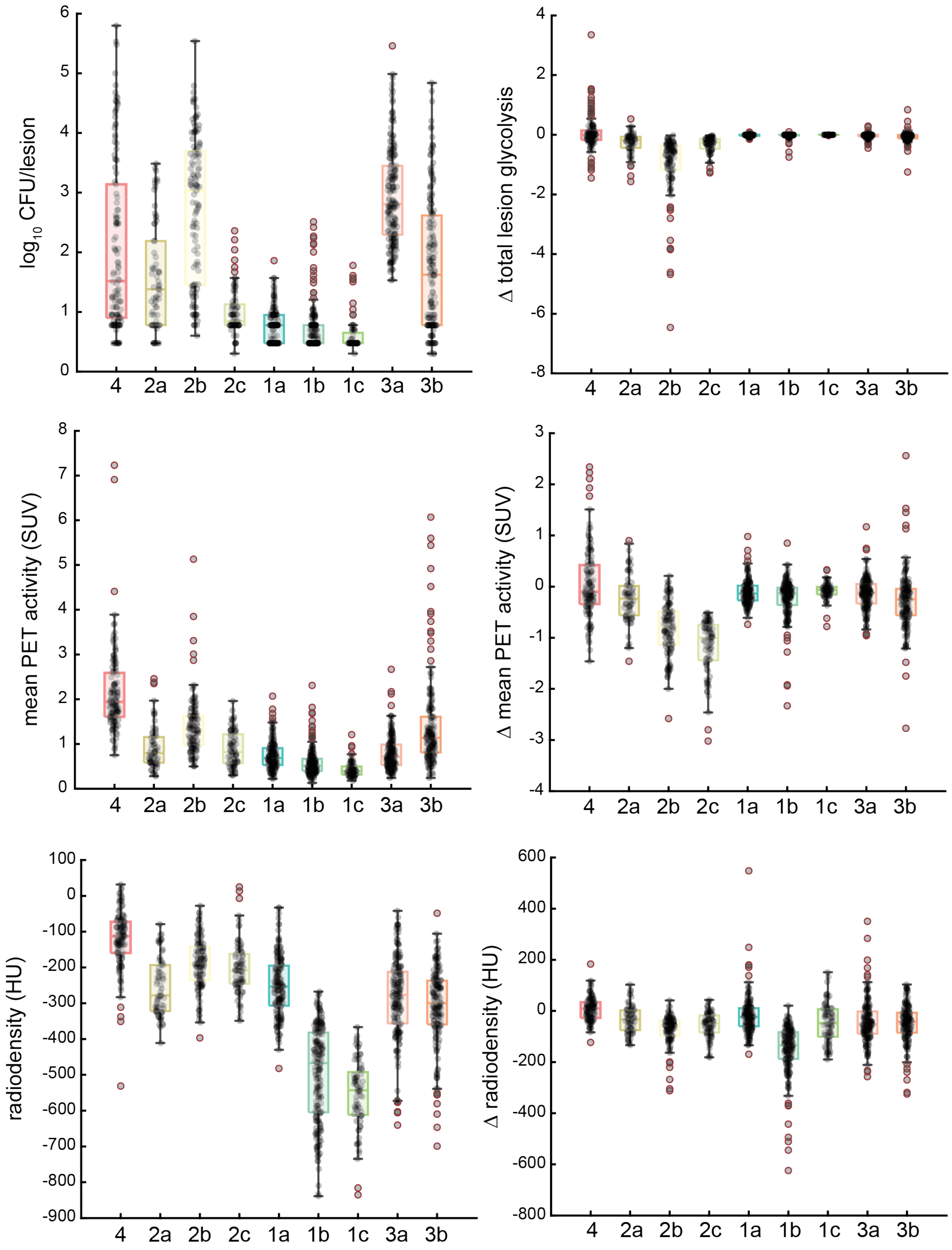

(continued on next)

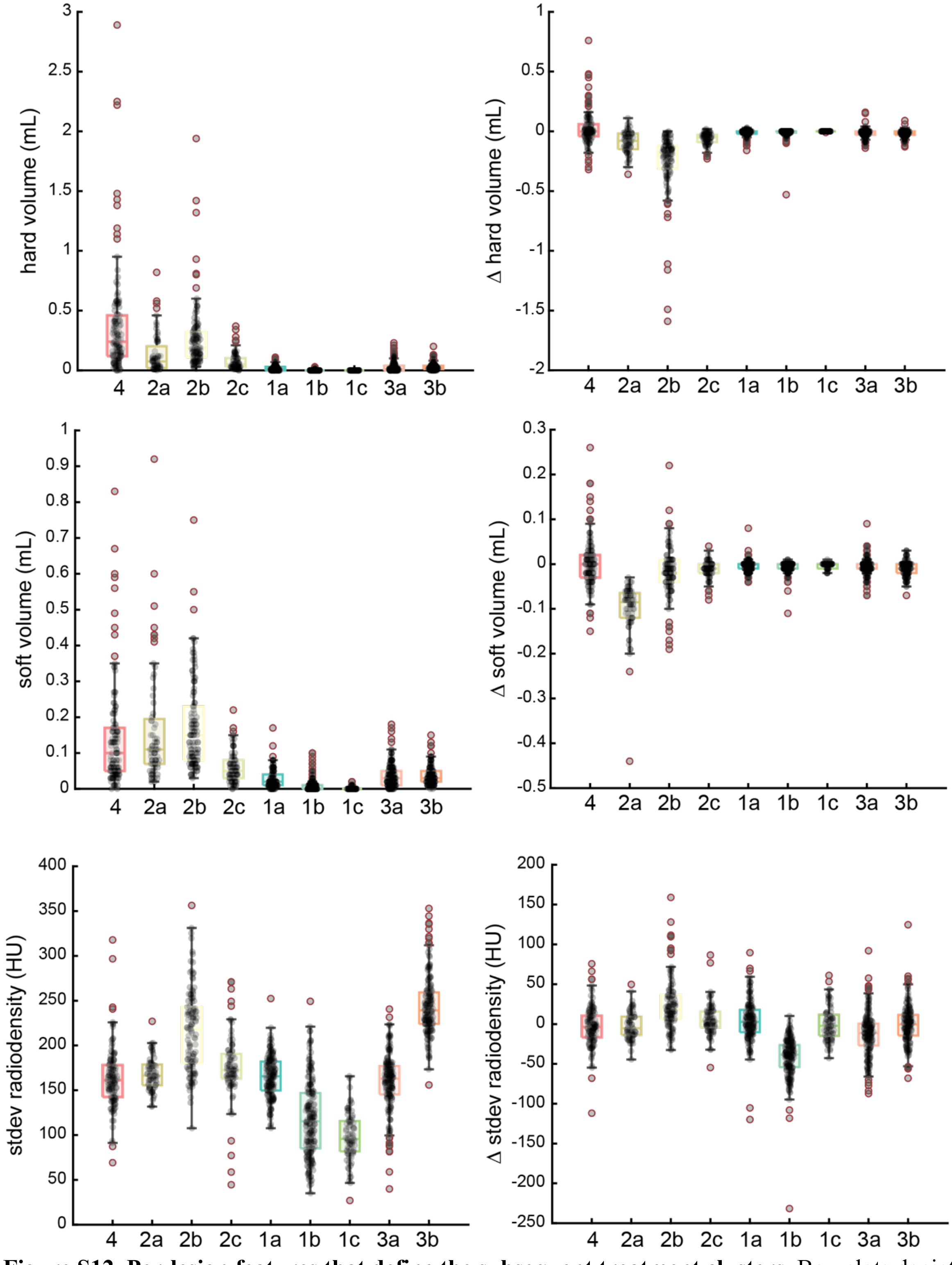

Figure S12. Per-lesion features that define the subsequent treatment clusters. Box plots depict the per-lesion features that define each cluster. Red outlines are outliers, values more than 1.5x IQR

(interquartile range). Δ refers to changes in features from the middle (4 weeks) to EOT (8 weeks). Cluster labels correspond to those defined in Figure 3.

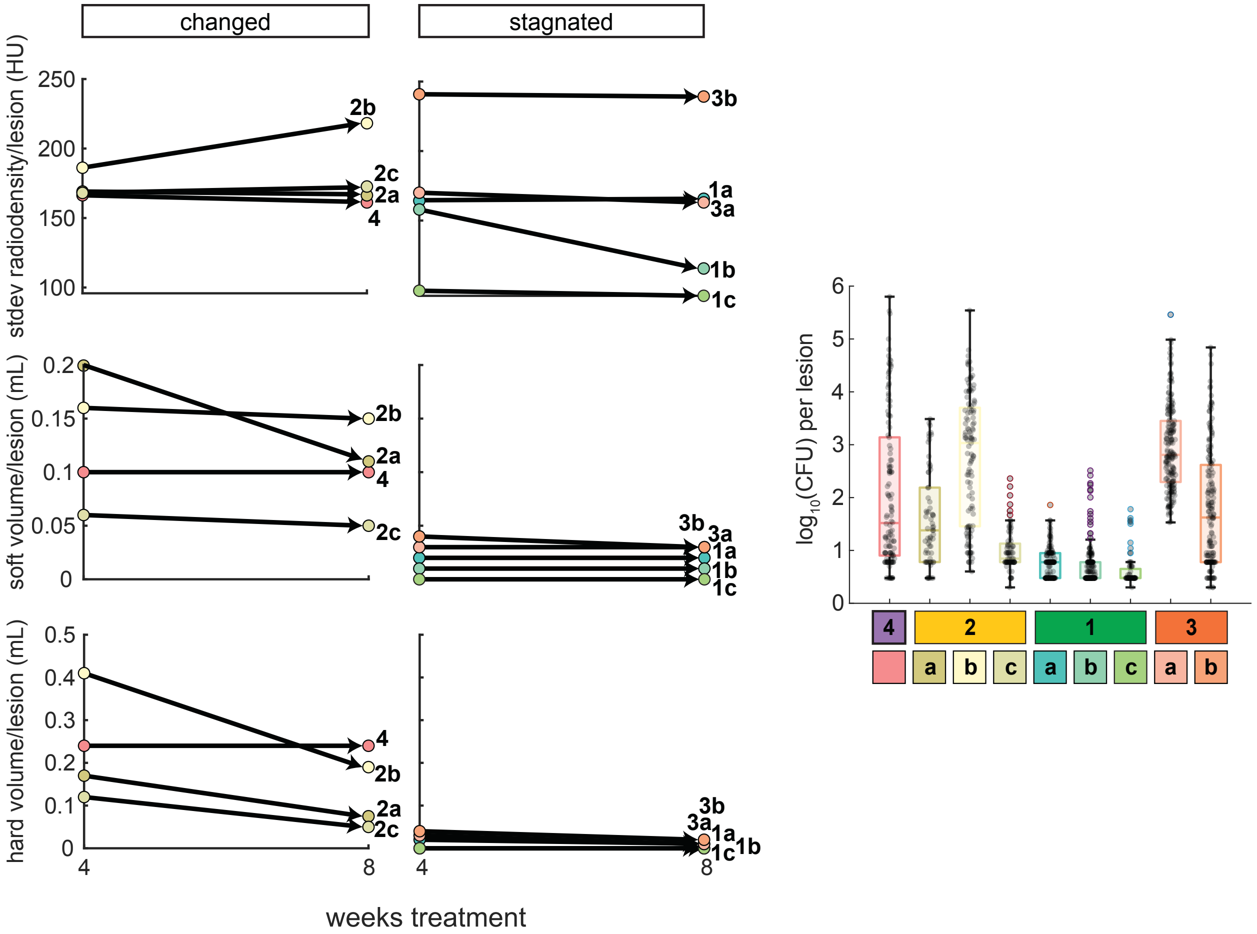

Figure S13. Additional features that define the continuation phase clusters. (A) Median feature values that characterize each lesion cluster at the middle (4 weeks) and end (8 weeks) of treatment and per-lesion bacterial burden distribution at necropsy across clusters. Colors correspond to those defined in Figure 3.

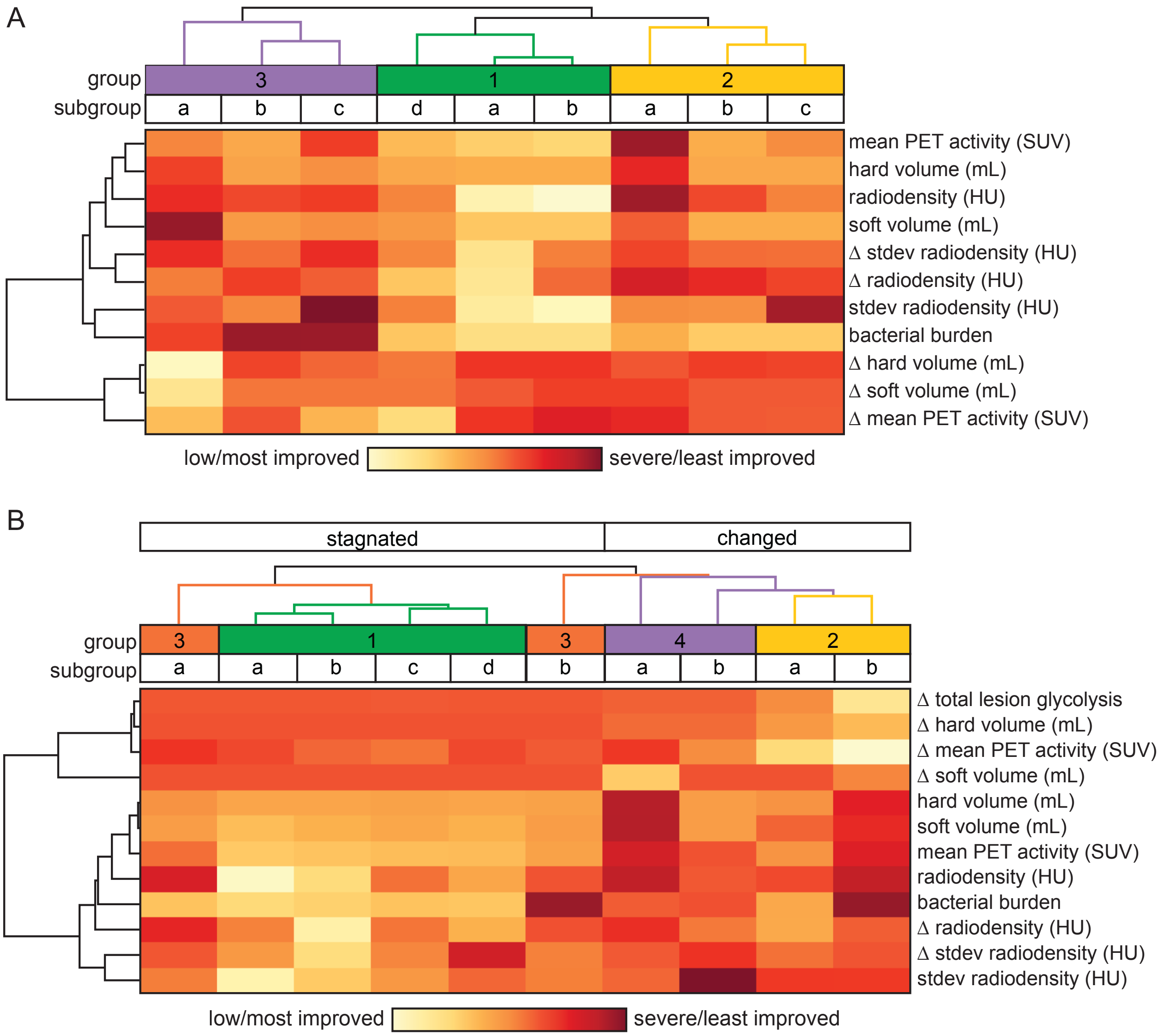

Figure S14. Feature profiles for lesion clusters from Leiden community detection with different time points. Clustering was performed using Leiden community detection using the terminal PET and CT features, bacterial burden, and change in features from after 2 weeks until EOT (8 weeks) or after 6 weeks of treatment until EOT. Median feature values across the resultant clusters were used in hierarchical clustering and displayed in heatmaps to identify patterns across the clusters similar to those identified in Figures 1 and 3. Feature values were scaled for visualization. The severe/low color scale is relative to the values in this visualization (not absolute). (A) Δ refers to changes in a feature from 2 weeks to EOT (8 weeks). (B) Δ refers to changes in a feature from 6 weeks to EOT (8 weeks).

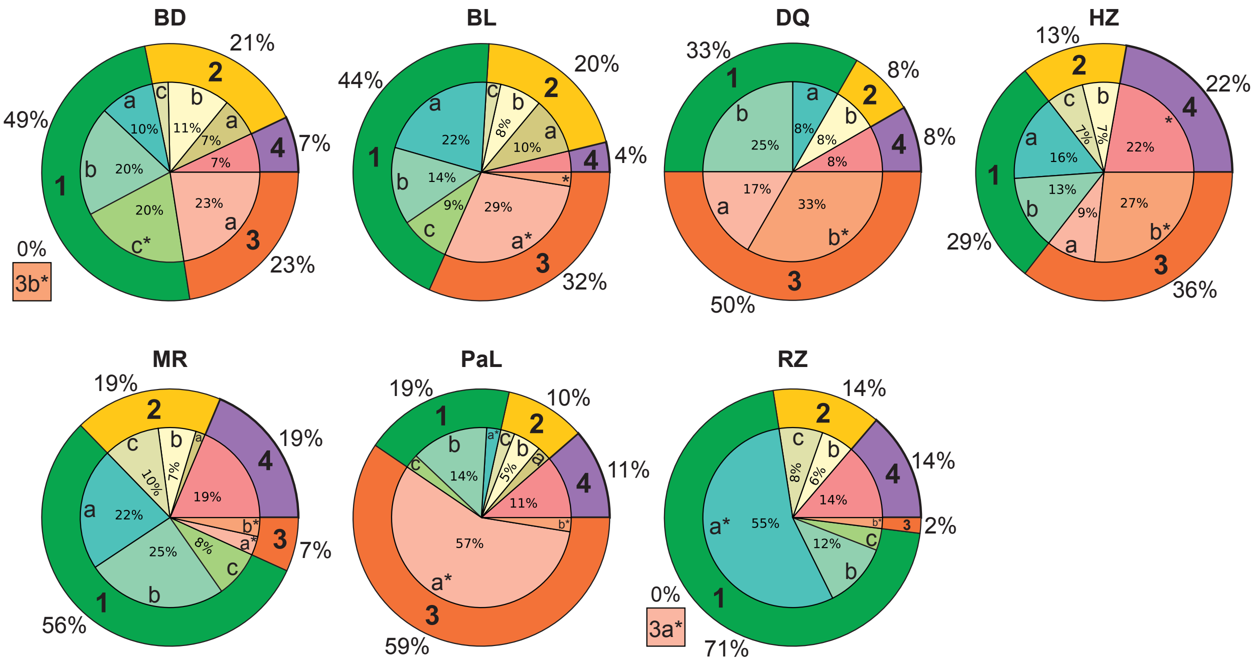

Figure S15. Distribution of subsequent treatment phase clusters across lesions from individual treatment arms. Inner pie charts represent the distribution of the clusters from the subsequent treatment phase (second half of treatment) analysis. Cluster groups are indicated in the outer pie charts. 1: low CFU and low PET/CT pathology with minimal improvement during subsequent treatment; 2: varied CFU and varied improvement in PET/CT pathology during subsequent treatment; 3: varied or high CFU and severe PET/CT pathology with minimal improvement during subsequent treatment; 4: varied CFU and severe PET/CT pathology that did not improve or worsened during subsequent treatment. Wedge colors correspond to those in Figure 3. * indicates significant deviation (95% confidence interval using significance threshold of 1.96) from the expected (average) number of lesions in a specific cluster by adjusted residual analysis following the chi-squared test for homogeneity ($p < 0.001$). Boxes to the left of each pie chart indicate significant underrepresentation from specified clusters that are completely absent. Unlabeled inner slices are <5% of the total for that drug treatment.

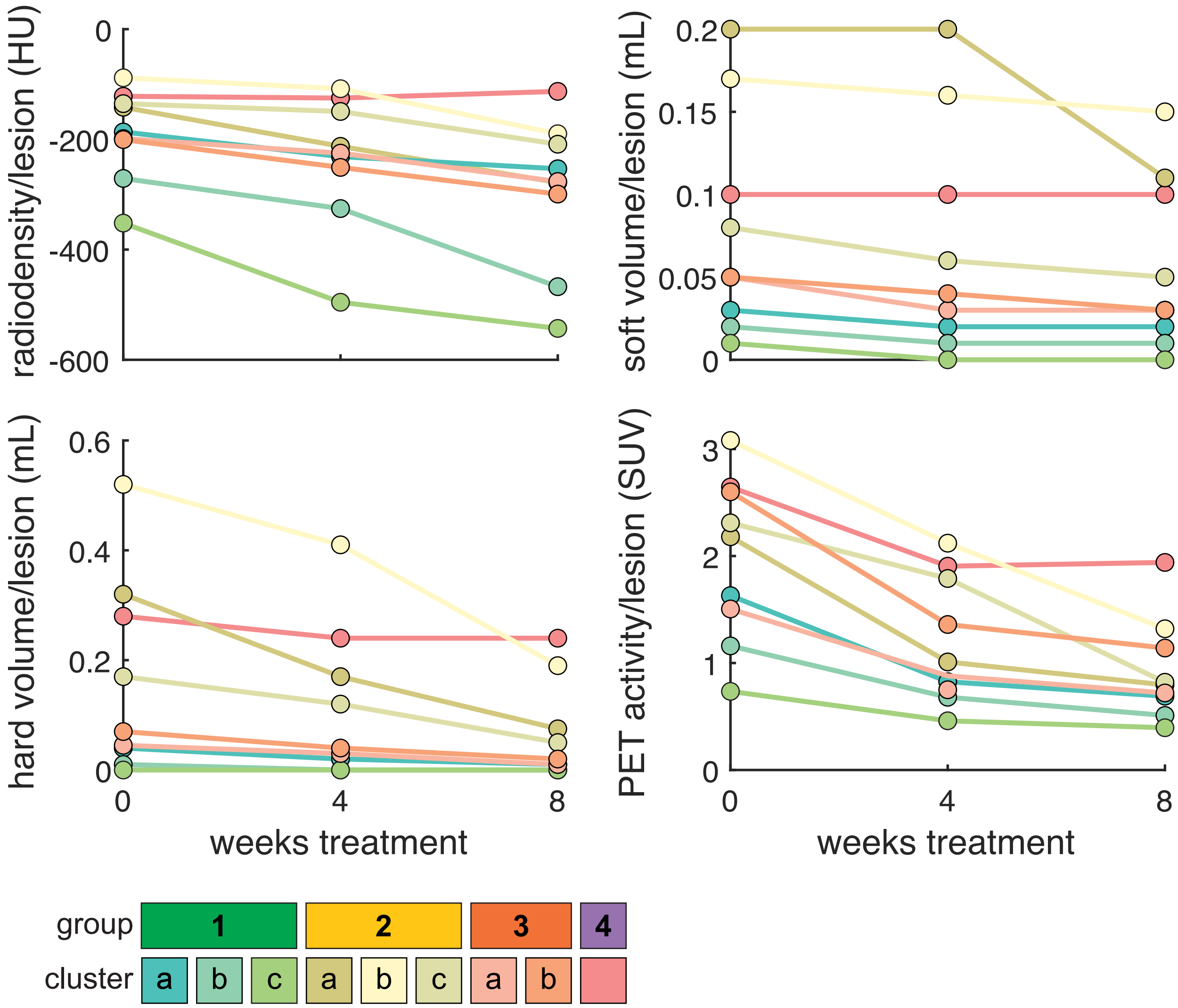

Figure S16. Continuation phase lesion cluster features at the start, middle, and end of treatment. Colors correspond to clusters and groups defined in Figure 3. Median feature values for lesions of each cluster from continuation phase analysis (i.e., lesion clusters defined based on terminal features and change in features during the continuation phase) at the start (0 weeks), middle (4 weeks, end of intensive phase/beginning of continuation phase), and end of treatment (8 weeks).

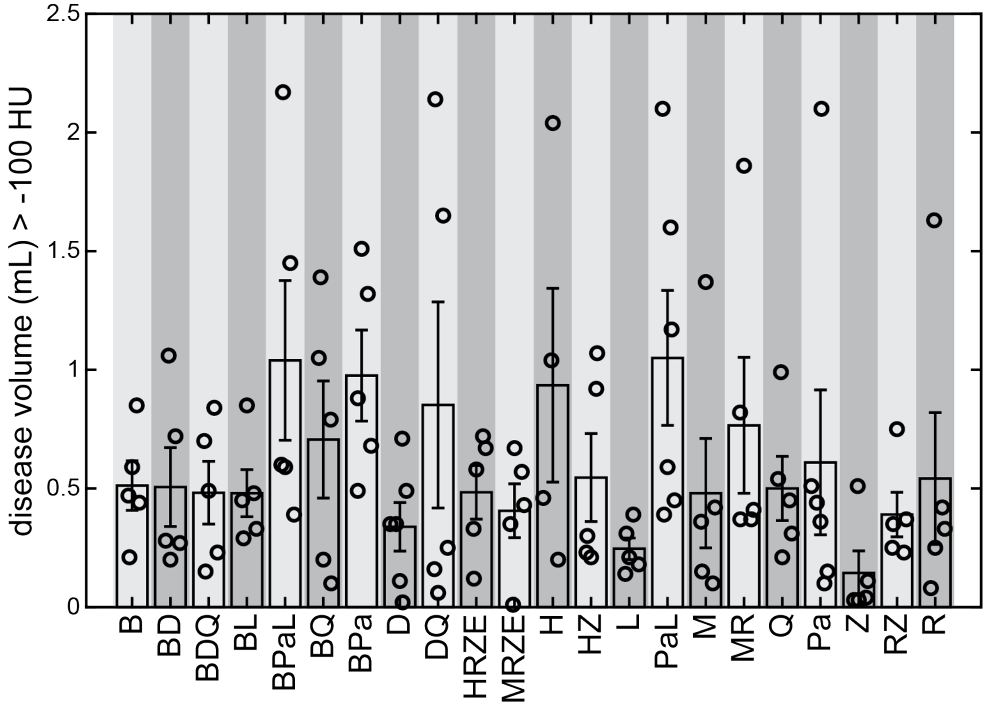

Figure S17. Distribution of total diseased lesion volumes across animals within treatment groups. Each bar represents the mean of total diseased (HU > -100) volumes across animals within each treatment group. Error bars represent the standard error within treatment groups. ANOVA was performed across all groups; $p = 0.22$.

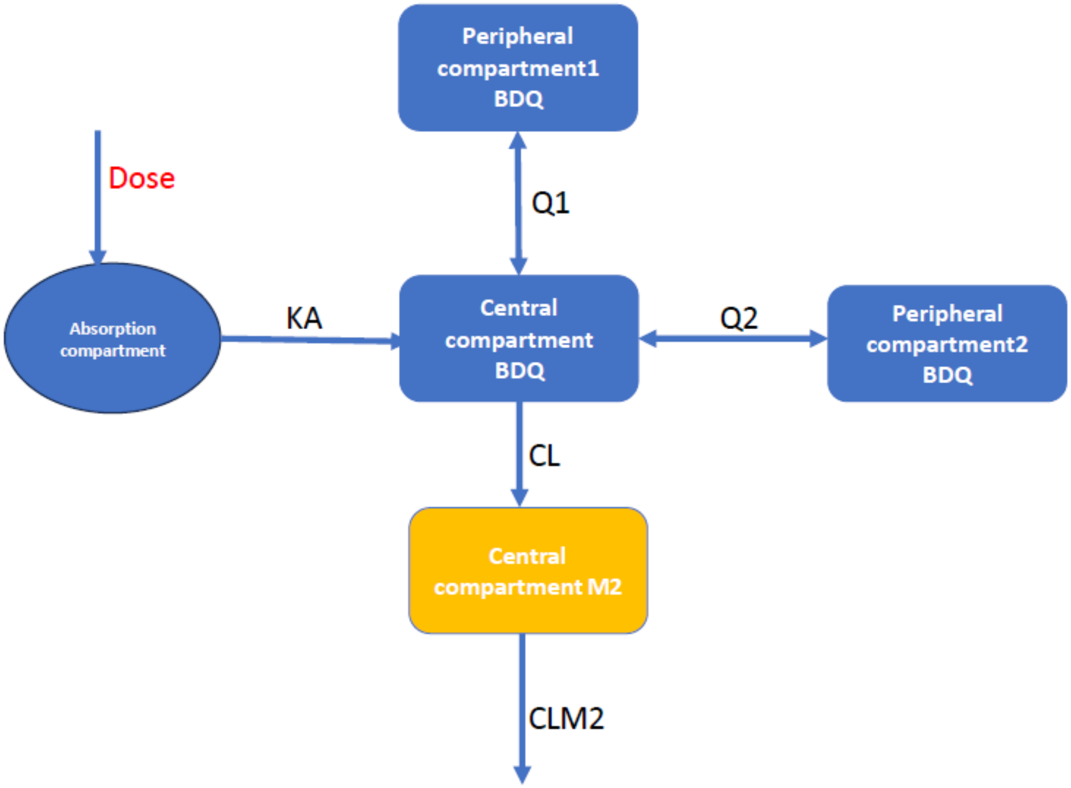

Figure S18. Parent-metabolite PK model scheme for bedaquiline and M2 following oral administration of bedaquiline in marmoset. BDQ: bedaquiline, CL: clearance of bedaquiline; CLM2, clearance of M2, KA: absorption rate constant, Q: Intercompartmental clearance.

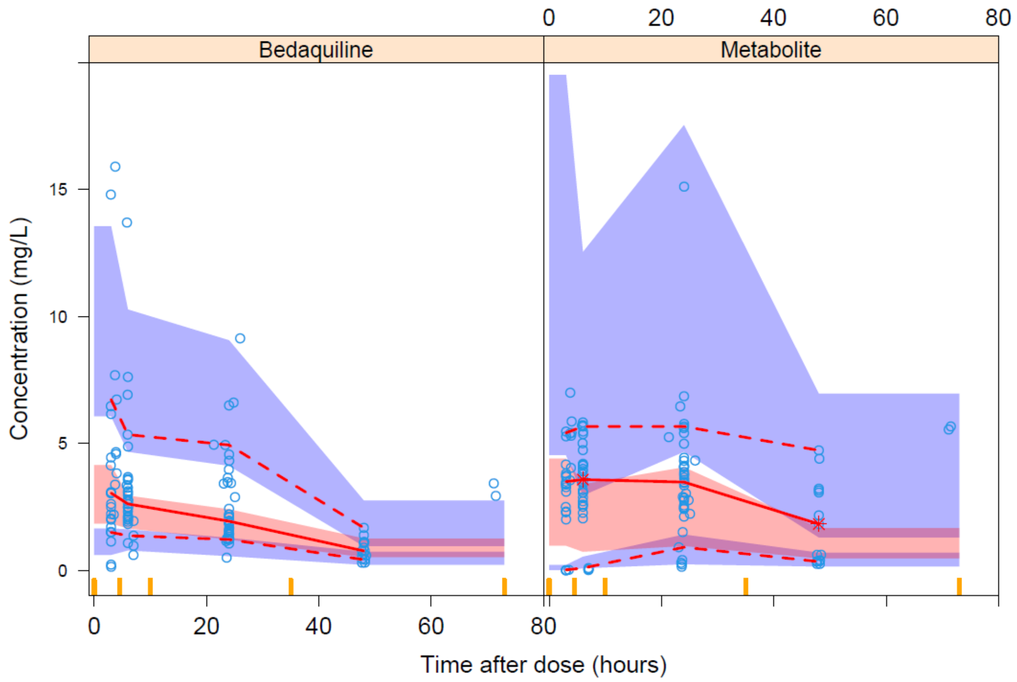

Figure S19. Visual predictive check (log scale). The lower, middle, and upper solid lines are the 10th, 50th, and 90th percentiles of the observed plasma concentration. The shaded areas are the 95% confidence intervals for the same percentiles, obtained from re-simulations of the same trial.

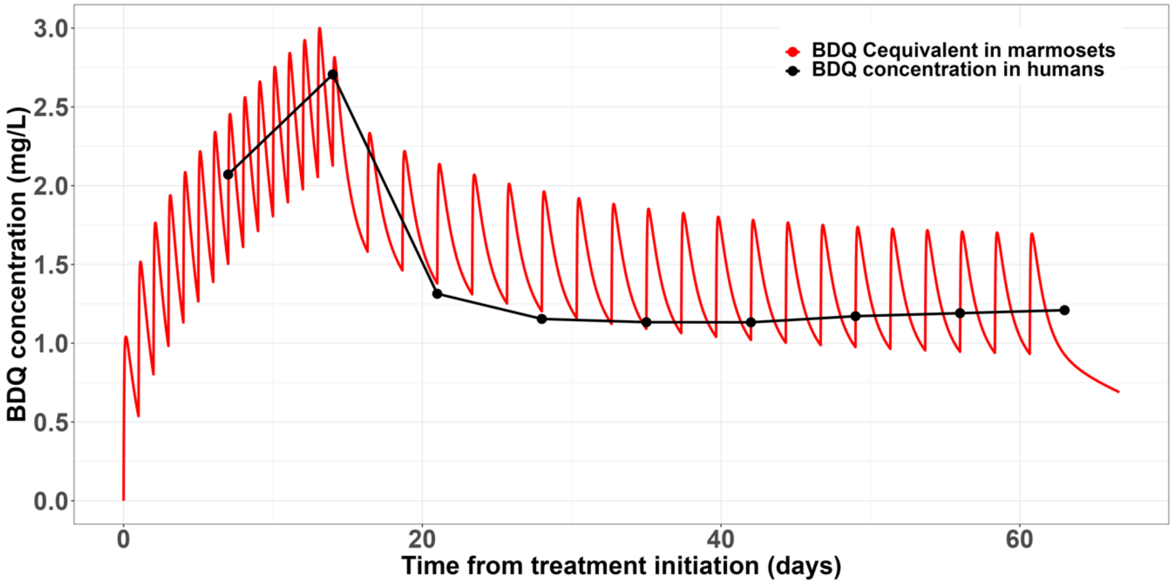

Figure S20. Prediction of the bedaquiline concentration vs. time curve in marmosets compared to humans. For marmosets, the chart shows Cequivalent (i.e., C_{BDQ} + CM2/5, to account for the bactericidal contribution of M2) obtained with a dosing regimen of 8 mg/kg daily for 2 weeks, followed by 6 mg/kg daily. For humans, the chart shows the concentration of bedaquiline alone (the bactericidal contribution of M2 is negligible in humans) using the standard regimen as reported by Svensson et al. (*38*).

Table S1.
Total number of lesions per animal and across all animals of each treatment arm used in analyses.

treatment arm	total # lesions/arm	# lesions per animal
bedaquiline (B)	62	8, 12, 17, 10, 15
delamanid (D)	46	10, 9, 6, 8, 5, 8
isoniazid (H)	34	6, 5, 16, 7
linezolid (L)	49	10, 8, 13, 8, 10
moxifloxacin (M)	61	15, 9, 11, 11, 15
quabodepistat (Q)	57	12, 7, 13, 12, 13
pretomanid (Pa)	43	9, 6, 11, 12, 5
pyrazinamide (Z)	45	8, 5, 11, 10, 11
rifampicin (R)	39	8, 9, 6, 9, 7
BD	71	11, 9, 16, 13, 22
BL	79	19, 12, 20, 13, 15
BQ	48	9, 5, 14, 10, 10
BPa	62	10, 14, 10, 14, 14
DQ	36	11, 10, 7, 4, 4
HZ	45	4, 12, 15, 9, 5
PaL	79	13, 27, 15, 10, 14
MR	59	11, 15, 10, 12, 11
RZ	51	13, 7, 10, 13, 8
DBQ	66	19, 14, 11, 10, 12
BPaL	72	16, 13, 17, 10, 16
HRZE	40	6, 5, 9, 13, 7
MRZE	49	10, 11, 10, 9, 9

Table S2.
Table of median values and standard deviation for each feature within each cluster described in Figure 1.

1			2			3			
**a**	**b**	**c**	**a**	**b**	**c**	**a**	**b**	**c**	
-143.86 ± 72.52	-239.33 ± 87.77	-89.29 ± 48.87	-121.11 ± 86.53	-71.18 ± 77.61	-76.37 ± 69.46	-112.86 ± 74.92	-57.90 ± 77.33	-0.64 ± 79.77	Δ(mean radiodensity) (HU)
-397.96 ± 98.54	-509.43 ± 124.82	-376.60 ± 120.00	-309.40 ± 92.37	-235.19 ± 88.71	-279.06 ± 100.88	-197.30 ± 71.10	-238.41 ± 113.22	-131.85 ± 71.78	mean radiodensity (HU)
0.55 ± 0.18	0.49 ± 0.21	0.50 ± 0.43	0.62 ± 0.33	0.77 ± 0.37	1.09 ± 0.44	1.19 ± 0.61	1.08 ± 1.11	1.71 ± 0.76	mean PET activity (SUV)
-45.98 ± 24.91	-51.25 ± 37.06	-15.10 ± 25.76	-5.32 ± 32.73	1.59 ± 44.24	16.06 ± 33.26	26.81 ± 33.36	4.06 ± 45.75	10.68 ± 30.28	Δ(stdev radiodensity) (HU)
117.19 ± 26.56	113.85 ± 42.95	124.83 ± 35.27	173.34 ± 35.07	168.45 ± 38.90	239.26 ± 26.51	183.80 ± 44.16	185.36 ± 50.02	164.63 ± 24.70	stdev radiodensity (HU)
0.00 ± 0.00	0.00 ± 0.00	0.00 ± 0.01	0.02 ± 0.08	0.03 ± 0.04	0.02 ± 0.04	0.26 ± 0.28	0.03 ± 0.13	0.14 ± 0.41	hard volume (mL)
0.01 ± 0.02	0.00 ± 0.02	0.01 ± 0.02	0.06 ± 0.06	0.03 ± 0.03	0.03 ± 0.03	0.18 ± 0.16	0.04 ± 0.04	0.07 ± 0.11	soft volume (mL)
2.63 ± 0.73	0.48 ± 0.44	0.48 ± 0.23	1.55 ± 1.07	0.85 ± 0.56	0.78 ± 0.45	2.03 ± 1.34	3.09 ± 0.80	1.00 ± 1.19	bacterial burden (log_{10}CFU)
-0.67 ± 0.45	-0.53 ± 0.61	-0.30 ± 0.40	-1.21 ± 0.72	-1.81 ± 0.91	-1.35 ± 0.73	-1.50 ± 1.04	-0.76 ± 0.72	-0.27 ± 0.73	Δ(mean PET activity) (SUV)
-0.01 ± 0.04	-0.01 ± 0.04	0.00 ± 0.01	-0.21 ± 0.20	-0.06 ± 0.07	-0.04 ± 0.06	-0.44 ± 0.38	-0.03 ± 0.07	-0.01 ± 0.20	Δ(hard volume) (mL)
-0.02 ± 0.02	-0.01 ± 0.02	-0.01 ± 0.01	-0.10 ± 0.06	-0.02 ± 0.03	-0.02 ± 0.02	-0.01 ± 0.09	-0.01 ± 0.03	0.00 ± 0.06	Δ(soft volume) (mL)

Table S3.

Table of median values and standard deviation for each feature within each cluster described in Figure 3.

4	2			1			3		
	**a**	**b**	**c**	**a**	**b**	**c**	**a**	**b**	
-0.00 ± 0.57	-0.17 ± 0.37	-0.65 ± 1.05	-0.27 ± 0.27	-0.01 ± 0.03	-0.01 ± 0.08	0.00 ± 0.01	-0.02 ± 0.08	-0.05 ± 0.16	Δ(total lesion glycolysis)
0.00 ± 0.15	-0.08 ± 0.09	-0.19 ± 0.26	-0.05 ± 0.05	-0.01 ± 0.03	0.00 ± 0.04	0.00 ± 0.00	-0.01 ± 0.04	-0.02 ± 0.03	Δ(hard volume) (mL)
-0.10 ± 0.70	-0.24 ± 0.48	-0.79 ± 0.53	-1.00 ± 0.54	-0.14 ± 0.24	-0.13 ± 0.37	-0.07 ± 0.16	-0.12 ± 0.31	-0.25 ± 0.56	Δ(mean PET activity) (SUV)
0.00 ± 0.06	-0.09 ± 0.06	-0.02 ± 0.06	-0.01 ± 0.02	0.00 ± 0.01	-0.01 ± 0.01	0.00 ± 0.01	-0.01 ± 0.02	-0.01 ± 0.02	Δ(soft volume) (mL)
161.21 ± 34.02	166.32 ± 18.32	218.20 ± 46.28	172.65 ± 37.68	165.64 ± 22.93	115.50 ± 41.76	95.83 ± 26.95	163.04 ± 31.60	239.22 ± 33.47	stdev radiodensity (HU)
1.52 ± 1.43	1.38 ± 0.89	3.03 ± 1.22	0.85 ± 0.39	0.78 ± 0.28	0.48 ± 0.39	0.48 ± 0.30	2.81 ± 0.81	1.62 ± 1.11	bacterial burden (log_{10}CFU)
1.94 ± 0.93	0.80 ± 0.49	1.32 ± 0.66	0.82 ± 0.39	0.69 ± 0.32	0.51 ± 0.31	0.40 ± 0.18	0.72 ± 0.38	1.14 ± 0.99	mean PET activity (SUV)
0.24 ± 0.44	0.08 ± 0.17	0.19 ± 0.28	0.05 ± 0.08	0.01 ± 0.02	0.00 ± 0.00	0.00 ± 0.00	0.01 ± 0.04	0.02 ± 0.03	hard volume (mL)
0.10 ± 0.14	0.11 ± 0.16	0.15 ± 0.12	0.05 ± 0.04	0.02 ± 0.03	0.01 ± 0.02	0.00 ± 0.00	0.03 ± 0.03	0.03 ± 0.03	soft volume (mL)
-112.91 ± 81.93	-278.29 ± 81.02	-189.79 ± 72.17	-208.76 ± 73.13	-253.34 ± 82.64	-467.29 ± 132.54	-543.05 ± 97.28	-276.47 ± 114.88	-299.28 ± 109.01	mean radiodensity (HU)
-3.78 ± 25.27	-5.28 ± 17.60	18.44 ± 33.42	3.23 ± 20.23	2.64 ± 25.71	-38.55 ± 25.45	-2.30 ± 21.52	-11.30 ± 27.59	-0.44 ± 24.32	Δ(stdev radiodensity) (HU)
9.84 ± 45.79	-32.53 ± 54.13	-58.22 ± 57.03	-48.64 ± 47.88	-23.47 ± 75.30	-134.62 ± 95.42	-47.84 ± 76.43	-52.73 ± 82.75	-41.73 ± 73.48	Δ(mean radiodensity) (HU)

Table S4.
Table of agent doses and formulations used in the marmosets and the range of steady-state exposure achieved.

Drug[a]<br>Source	Marmoset Dose<br>(for 2 months)	Oral formulation for marmosets[e]	$AUC_{(0-24h)}$[f]<br>(ug*h/mL) range
Linezolid (L)<br>AmBeed, Arlington Hts, IL	20 mg/kg daily	powder in 10% (v/v) water and 90% (v/v) OraSweet[d](v/v) and 2% flavor (v/v)	140-280
Pretomanid (Pa)<br>Bioduro LLC, Irvine, CA	5 mg/kg daily	in 20% (w/v) Captisol, 50% (v/v) OraSweet, 49% (v/v) water-saccharin (20% w/v saccharin) and 1% (v/v) flavoring	33-41
Bedaquiline (B)[b]<br>Janssen Pharmaceuticals<br>jnj.com	8 mg/kg x 14 d, 6 mg/kg, 3 x wk	100 mg USP Sirturo tablets powdered and suspended in a syrup composed of 1:1 OraPlus/OraSweet[d] (v/v) and 2% flavor (v/v)	33-64<br>C_{avg}: 1.7 ± 0.5 µg/mL SD
Delamanid (D)[c]<br>Otsuka PDC, Rockville MD	10 mg/kg daily	in 20%(w/v) Captisol (Ligand Pharmaceuticals, San Diego CA), 50% (v/v) OraSweet, 49% (v/v) water-saccharin (20% w/v) 1% v/v flavor, pH 3	2-7
Quabodepistat (Q)<br>Otsuka PDC	7.5 mg/kg daily	powder in 50% (v/v) OraPlus, 48% (v/v) OraSweet and 2% (v/v) flavor.	3-17
Moxifloxacin (M)<br>LKT Labs<br>St Paul, MM	45 mg/kg daily	powder in 94% (v/v) H_20 + DexSacch (5% (w/v) Dextrose and 15% (w/v) Saccharin) and 4% (v/v) flavor	16-53<br>(Median 34)
[a] Isoniazid (USP tablets powdered, Mylan.com); Rifampicin (USP Rifadin IV, Sanofi, Bridgewater NJ) Pyrazinecarboxamide (Sigma Aldrich, St Louis MO) and Ethambutol (Ethambutol dihydrochloride, Sigma Aldrich, St Louis MO), dosed daily, and had AUCs as previously described *(13)*.			
[b] Given 30 min after a daily dose of 1-aminobenzotriazole 20 mg/kg (Cayman chemical, Ann Arbor, MI); dosing was after feeding with fat supplementation; Des-bedaquiline metabolite C_{avg}: 2.5 µg/mL ± 0.49 µg/mL. x			
[c] Delamanid hot melt granules and spray-dried formulations (Otsuka PDC, Rockville MD). Given 30 min after other drugs when given in combination with quabodepistat and bedaquiline			
[d] OraPlus/OraSweet and flavoring (Perrigo, Grand Rapids MI)			
[e] Agent powders or granules were suspended in the formulation first with a mortar and pestle, then mixed with sweeteners & flavors followed by bath sonication until homogeneous.			
[f] Drug monitoring during treatment did not reveal altered exposure of the single drugs when given in combination, except for quabodepistat in combination with bedaquiline or bedaquiline+delamanid where the exposure was increased by 3 to 18X the individual dose. For this reason, when Q was combined with B, Q was also given 3 x wk after the 14-day loading period.			

Table S5.

Pharmacokinetic parameter estimates of the structural and final model in marmosets.

Fixed effects	Value	Random effects	Value
CL (L/h)	0.046	BSV CL (%)	30.4
V (L)	0.539	BSV CLM2 (%)	78.4
KA (1/h)	0.204	BOV F (%)	53.5
F (.)	1 FIX	BOV KA (%)	80.7
LAG (h)	1.57	BOV LAG (%)	40.2
Vp1 (L)	9.96		
Vp2 (L)	1.53	Prop error BDQ (%)	11.7
Q1 (L/h)	0.0729	Prop error M2 (%)	12.7
Q2 (L/h)	0.392		
CLM2 (L/h)	0.0362		
VM2 (L)	4.2		

BDQ, bedaquiline; CL, bedaquiline clearance; V, central compartment volume; KA, first-order absorption rate constant; F, oral bioavailability; LAG, absorption lag time; Vp1 and Vp2, volume of distribution for peripheral compartments; Q1 and Q2, intercompartmental clearances; CLM2, M2 clearance; VM2, M2 central compartment volume. Typical values of clearance and volume of distribution were allometrically scaled with body weight, and the typical values reported are for a marmoset with a body weight of 0.41 kg (median of all study marmosets). BSV, between subject variability; BOV, between occasion variability.
